# Breast Cancer Brain Metastases: Current Understanding and Future Directions

**DOI:** 10.1007/s11912-026-01753-y

**Published:** 2026-02-05

**Authors:** Mohammad Alabdulrahman, Gordon R. Daly, Sindhuja Naidoo, Lea Stuart, Sami Almasri, Ali Benour, Jason McGrath, Minatoullah Habaka, Gavin P. Dowling, Cian M. Hehir, Arnold DK. Hill, Damir Varešlija, Leonie S. Young

**Affiliations:** 1 The Department of Surgery, RCSI University of Medicine and Health Sciences, Dublin, Ireland; 2 The Department of Surgery, Beaumont Hospital, Dublin, Ireland; 3 School of Pharmacy and Biomolecular Sciences, RCSI University of Medicine and Health Sciences, Dublin, Ireland; 4 Beaumont RCSI Cancer Centre, Beaumont Hospital, Dublin, Ireland

**Keywords:** Breast cancer brain metastases, Receptor discordance, Cancer neuroscience, Spatial transcriptomics, Liquid biopsies, Radiomics, Machine learning, PI3K/AKT/mTOR, Homologous recombination deficiency, JAK/STAT3, C-MYC, RET, HER3, Astrocyte-tumour interactions

## Abstract

**Purpose of Review:**

To examine recent advances in understanding breast cancer brain metastases (BCBM), with emphasis on metastatic mechanisms, tumour-microenvironment interactions, receptor discordance between primary breast tumours and brain metastases, diagnostic innovations, and therapeutic strategies. The review sought to clarify how these developments inform precision management and identify priorities for future research.

**Recent Findings:**

Studies implicate JAK–STAT signalling, homologous recombination deficiency, *c-MYC*, PI3K/AKT/mTOR, and *RET* in BCBM progression. Tumour cells adapt via neuronal mimicry, metabolic reprogramming, and crosstalk with astrocytes and microglia. Receptor discordance between primary breast cancers and brain metastases occurs in up to one-third of cases and may alter systemic therapy. Emerging tools including liquid biopsy, spatial transcriptomics, and radiomics offer minimally invasive approaches for molecular profiling, spatial mapping, and imaging-based phenotyping to guide personalised management. Machine learning supports prognostication and imaging interpretation, though external validation is limited.

**Summary:**

BCBM reflect complex tumour-brain interactions. Advances in molecular understanding and diagnostics are beginning to inform the development of targeted therapies. Future work may benefit from integrating cancer neuroscience with computational modelling, standardise diagnostic platforms, and test the survival impact of receptor reassessment in prospective trials.

## Introduction

Brain metastases occur in up to 40% of patients with breast cancer and remain a major cause of morbidity and mortality despite advances in systemic control and neuroimaging [1, 2]. Breast cancer brain metastases (BCBM) present unique clinical and biological challenges, arising from both the intrinsic aggressiveness of certain breast cancer subtypes and the distinct selective pressures of the central nervous system microenvironment. Once tumour cells reach the brain, they must adapt to its specialised neural and vascular niche, remodel the blood brain barrier, and engage in dynamic interactions with astrocytes, microglia, and neurons. These processes are increasingly recognised within the growing field of cancer neuroscience. Such adaptations are driven by diverse oncogenic pathways, genomic instability, and changes in receptor status, which together influence both disease progression and therapeutic response. At the same time, novel diagnostic approaches such as liquid biopsy and spatial transcriptomics, together with evolving systemic and local treatments including targeted agents, immunotherapy, and brain penetrant drugs, have the potential to influence management strategies, although their clinical roles remain under evaluation. This review synthesises current knowledge of metastatic mechanisms, receptor discordance, diagnostic advances and treatment developments, while examining how computational modelling and neurobiological insights may work together to guide future precision strategies in BCBM.

## Established Metastatic Pathways

### Plasticity and Microenvironment Interactions

#### JAK/STAT Pathway

The Janus kinase/signal transducer and activator of transcription is an important regulator of the microenvironment surrounding BCBM. Under normal conditions, the JAK/STAT3 pathway acts in promoting the activation of A2 reactive astrocytes (RA), which are implicated in scar-formation following ischemia [3]. This pathway is also involved in the initiation of astrogliogenesis and subsequently the maturation of astrocytes during developmental stages of the brain [3]. The role of JAK/STAT3 in metastasis has been a topic of exploration across recent studies such as that of Priego et al., who demonstrated the elevated expression of activated STAT3 and its promotion of metastases in both human and mouse samples [4]. The secretomes of these STAT3 expressing RAs include several immunosuppressive molecules such as vascular endothelial growth factor-A (VEGF-A), lipocalin-2, programmed cell death ligand 1 (PD-L1), and tissue inhibitor of metalloproteinases-1 (TIMP-1) [4]. Taken together with the observed reinforcement of the extracellular matrix of these cells, they may contribute to an immunosuppressive microenvironment that impedes immune cell access, particularly to the access of CD8 + cytotoxic T cells [4]. Moreover, STAT3 + RAs demonstrate elevated expression of CD74 + macrophage migration inhibitory factor (MIF) and increased binding to CD74 + microglia, establishing a form of “cross-talk” between microglia and RAs, which results in the upregulation of the nuclear factor (NF)–κB signalling pathway and supporting an environment permissive to metastatic progression in experimental models [5]. 

### DNA Repair and Genomic Instability

#### Homologous Recombination Repair Deficiency (HRD)

The homologous recombination repair pathway (HRR), is a complex pathway, comprised of multiple genes that contribute to high-fidelity repair of damaged DNA. HRD and has been linked with the development of brain metastasis and shown to be more prevalent when brain metastasis develops. Abnormalities within this pathway, particularly relating to repair genes such as *BRCA1/2*, *PALB2*, *RAD51C*, *ATM*, *CHEK2* and many other genes, have been associated with an increased risk of developing breast cancer [6, 7]. HRD status has crucial implications for therapeutic response to both PARPis and platinum-based chemotherapies [8, 9]. 

Studies have reported increased levels of HRD in BCBM compared to primary breast cancer and that HRD may be present independent of somatic and germline mutations in HRD pathway genes [10, 11]. These studies highlight two plausible causes for this phenomenon: (1) clonal selection of primary tumour cells with higher HRD scores, the greater the instability, the more potential for the cells to adapt to new microenvironments within the brain; (2) genomic instability develops due to an unidentified mechanism, as the metastases develop [10, 11]. 

These studies highlight the presence of HRD in BCBM, independent of primary tumour mutations, and the role that HRD plays in the progression of breast cancer to the brain. Further research into the gene abnormalities within the HRR pathway, will provide an opportunity for novel therapeutics, to be used and developed based on the deficiencies and abnormalities of genes within the HRR pathway [12]. Recent studies explore therapeutic agents that target some of the known mutations which have been evaluated in clinical trials for their efficacy in HRD-positive BCBM [13]. 

The OlympiAD trial has successfully highlighted the benefit of olaparib and talazoparib, both of which are PARP inhibitors (PARPis) that target HRD-positive metastatic breast cancer, in patients with *BRCA1/2* mutations [14]. Similarly, the EMBRACA trial explored the overall survival of patients with metastatic breast cancer, treated with talazoparib vs. chemotherapy. Talazoparib is approved in the US, EU, and other countries for Human Epidermal growth factor Receptor 2-negative (HER2-), locally advanced or metastatic breast cancer with BRCA1/2 mutations [15]. Newer PARPis such as AZD9574 and pamiparib have exhibited increased BBB penetrance in pre-clinical in vivo studies promoting increased drug deliverance, while reducing toxic side effects. These findings suggest potential for future clinical application, pending validation [16, 17]. 

Current ongoing clinical trials such as NCT06458712 are investigating the impact of DSB2455 monotherapy in patients with HRD positive advanced malignancies, including BCBM [18]. DSB2455 is a PARP inhibitor with strong CNS penetrance, the results of this clinical trial are expected to clarify whether CNS-penetrant PARPis offer clinical benefit [18]. Research on the effects of individual and/or combined targeted therapies on BCBM is important to continue expanding effective treatment options for patients [10]. Further areas of interest include: novel agents targeting the HRR repair pathways, such as *ATR* and *WEE1* inhibitors [19]. Ongoing research to determine their potential clinical applicability to brain metastases has not yet been addressed but holds promise for future application in BCBM [19]. 

### c-MYC

The MYC gene, specifically the *c-MYC* isoform, plays an important role in breast cancer progression and metastasis, including to the brain. Because of its role as a transcription factor, *c-MYC* regulates genes involved in cell proliferation, metabolism, and apoptosis [20]. Overexpression of *c-MYC* is often observed in aggressive breast cancers and is associated with poor prognosis. In the context of brain metastases, *c-MYC* has been shown in preclinical models to enhance the invasive capacity of breast cancer cells, facilitating their colonization in the brain microenvironment [20]. 

Furthermore, *c-MYC* contributes to metabolic reprogramming of cancer cells, promoting aerobic glycolysis and lipid metabolism, which are essential for tumour growth and survival in the brain environment. This ‘reprogramming’ supports the energy demands of rapidly proliferating metastatic cells and aids in their adaptation to the brain niche [21]. Additionally, *c-MYC* influences the tumour microenvironment by promoting angiogenesis and immune evasion, further supporting metastatic progression [20, 21]. 

Interestingly, while *c-MYC* drives metastasis, it also renders brain-metastatic breast cancer cells more susceptible to certain therapies, such as TRAIL-induced apoptosis, suggesting a potential for therapeutic applications [22]. A recent cohort study of 42 BCBM samples were analysed using whole exome sequencing (WES) and total RNA sequencing and showed evidence of increased expression of MYC gene in BCBM samples in comparison to primary tumour samples [23].

A recent phase 1 clinical trial on the use of OMO-103, a MYC inhibitor, on solid tumours, including triple negative breast cancer (TNBC) was published [24]. This trial reported preliminary antitumour activity of OMO-103, including in combination with chemotherapy, immunotherapy and personalised approaches, citing the importance of continued investigation into the use of MYC inhibitors [24]. There are currently no preclinical investigations into the use of this targeted therapy in breast cancer, further specific studies into the use of MYC inhibitors is needed to gain insight into the potential clinical applicability in other BC subtypes and BCBM.

### ARID1A

AT-rich interactive domain-containing protein 1 A (ARID1A) codes for key proteins in the SWI/SNF chromatin remodelling complex, an important complex for gene expression and chromatin remodelling [25]. Inactivating mutations in *ARID1A* despite being found in only 2.5% of all breast cancers, are found in 12% of treatment resistant metastatic breast cancer suggesting a possible role in breast cancer metastasis [26]. The rationale behind this is that *ARID1A* regulates EMT activity; therefore, deactivation of *ARID1A* leads to increased cell proliferation, migration, invasion, angiogenesis, and reduces sensitivity to some chemotherapy [27]. 

Along with chemotherapy *ARID1A* is importantly implicated in the response to endocrine therapy via key interactions with various transcription factors. For instance, *ARID1A* helps maintain ER signalling, making it essential for the responsiveness of drugs.

that target oestrogen including tamoxifen [28]. Tamoxifen-bound ER primarily represses gene expression by binding to enhancer regions and signalling for *ARID1A* to recruit histone deacetylase 1 (HDAC1), an important histone-modifying enzyme that silences gene expression. When *ARID1A* is lost, HDAC1 recruitment fails, histone acetylation increases, and the chromatin remains active, leading to ER therapy resistance [26]. Thus, *ARID1A* mutations have been linked to more aggressive tumour behaviour, worsening prognosis and potentially a subsequent increased risk for brain metastasis.

Given these mechanistic insights, there is currently very limited research into therapies specifically targeting *ARID1A* enriched BCBM. However, several early-phase trials are underway with an ongoing clinical trial (NCT05490472), assessing the use of Aurora A kinase inhibitor JAB-2485 monotherapy in patients with advanced Estrogen Receptor-Positive (ER+) breast cancer, TNBC, and *ARID1A* mutant solid tumours [29]. Additionally, clinical trial (NCT04957615) is in phase II trial studies on the use of nivolumab on patients with metastatic malignant solid tumours and unresectable solid tumours with *ARID1A* mutations and CXCL13 expression [30]. While both studies include patients with brain metastases, there is a clear criteria of exclusion for patients with active progression of their brain metastases. The result of these ongoing clinical trials are expected to provide insight provide insight into the clinical relevance of targeted agents for the *ARID1A* mutation and their efficacy against cancers with this mutation in advanced solid tumours. There are currently no ongoing studies into the use of targeted therapeutics in specifically BCBM patients with *ARID1A* mutations. These trials may inform future studies evaluating whether targeted agents could benefit *ARID1A*-altered BCBM.

### Receptor-driven Targeted Axes

#### PI3K/AKT/mTOR

Another well-known pathway that is activated in 43–70% of patients with BCBM, is the PI3K/AKT/mTOR pathway [31]. PI3K is a lipid kinase family, with Class I being the most relevant in BCBM [32]. Upon the activation of PI3K, PIP2 converts to PIP3, which subsequently results in signalling via AKT and mTORC1 leading to cell growth and survival [32]. The mechanistic target of rapamycin (mTOR) functions as a critical oncogenic driver in multiple malignancies. As a serine/threonine kinase, mTOR integrates signals from growth factors, nutrients, and cellular energy status to regulate proliferation, protein synthesis, and survival [33]. In breast cancer, aberrant activation of the PI3K/AKT/mTOR axis, often due to *PIK3CA* mutations or *PTEN* loss, leads to constitutive mTOR signalling, enhancing metastatic potential [34]. In the brain microenvironment, where metabolic constraints and immune evasion are crucial for tumour survival, mTOR activity supports cellular adaptation and outgrowth [35]. Its role as an oncogene is further highlighted by evidence linking mTOR hyperactivation with poor prognosis and resistance to therapy [36]. These findings justify continued exploration of mTOR-targeted therapies, especially brain-penetrant inhibitors, in managing breast cancer brain metastases [31]. 

A preclinical study using WES of 86 matched BCBM samples, were analysed. Alterations in the PI3K/AKT/mTOR pathway was detected in the BCBM samples [37]. There were 43 alterations in the 37 cases that occurred in 10 of the total 15 genes that were evaluated [37]. 14 of the 43 alterations were detected only within the BCBM samples, and 24 were shared between primary tumour and BCBM, the remaining 5 were found only in the primary sample [37]. A further analysis of the four cases with PI3K mutations, showed that all metastatic sites shared the PIK3CA mutation and were treated with a PI3K inhibitor, with no evidence of intracranial disease progression for 8 months [37]. This was further supported in another preclinical study using dual PI3K/mTOR inhibitors (GDC-0084) and establishing their use as inhibitors of the metastatic process [31]. PIK3CA-mutant and wild type breast cancer cell lines, as well *as in vivo* GDC-0084 on BCBM xenograft mouse models were used [31]. This study highlighted the role of GDC-0084, which inhibited only the wild-type cell line in vitro, while in-vivo treatment showed significant inhibition of growth of the PIK3CA-mutant [31]. 

Furthermore, the recent development of MEN1611, an investigational drug agent that blocks the PI3K protein in cancer cells, has been investigated in clinical trials such as the B-Precise-01 trial. B-Precise-01 (NCT03767335) investigated the use of MEN1611 in combination with trastuzumab, with or without fulvestrant, for the treatment of metastatic or advanced HER2 + breast cancer. The results of this trial highlight the positive response against the HER2+/PIK3CA-mutated advanced or metastatic breast cancer, as well as the duration of response in this patient group [38]. Another clinical trial of note targeting the PI3K mutation is the SABINA trial which is currently an ongoing phase II clinical trial investigating the efficacy and safety of MEN1611 monotherapy or in combination with eribulin in HR/HER2 negative PIK3CA/PTEN-altered, unresectable advanced or metastatic metaplastic BC patients [39]. The results of this clinical trial will provide new insight into the PI3CA mutation and its role in BCBM, as well as the clinical significance of targeted therapies such as MEN1611.

#### RET

*RET* is a specific oncogene, with three main isoforms *RET9*, *RET51* and *RET43*. *RET9* and *RET51* have been expressed in multiple breast cancer cell lines, with varying degrees of expression and signal transduction [11]. *RET* has primarily been studied in ER + breast cancer, with ER activation inducing *RET* expression, thus driving cell proliferation [11]. 

A recent study assessed *RET* function in a cohort of patients with primary and brain metastatic tumours [40]. In vivo models, organoid models and brain organotypical cultures were used, with RNA sequencing [40]. This study showed a statistically significant level of *RET* in brain metastases in ER + breast cancer [40]. Subsequently *RET* overexpression augmented the processes in cell adhesion, using epidermal growth factor receptors (EGFR) to subsequently deliver a “pro-brain metastatic phenotype” [40]. This novel finding specifically with regard to the actions and functions of *RET* in BCBM, has shown the statistically significant role of the *RET* oncogene in regulating the colonization and outgrowth of BCBM. Gattelli et al. used in vivo inhibition of *RET* in a metastatic breast cancer model, showing inhibited tumour growth and metastatic potential [41]. These findings further supported the possible role of *RET* in the metastatic pathway of BCBM [41]. While the exact mechanisms are not completely understood in subtypes other than ER + breast cancer, there is significant evidence supporting the role of the *RET* oncogene in the metastatic pathway of breast cancer [41]. 

There are no current clinical trials that specifically investigate targeted therapies against BCBM with known *RET* mutations. The LIBRETTO-431 trial was conducted in patients with non-small cell lung cancer (NSCLC), 42 of the 192 patients had brain metastases. 82% of the patients with brain metastases who were given selpercatinib demonstrated intracranial response. selpercatinib is a highly selective, brain penetrant *RET*-kinase inhibitor [42]. Furthermore, patients with *RET* positive NSCLC that were treated with selpercatinib experienced longer progression-free survival in comparison to those receiving chemotherapy with or without pembrolizumab [42]. Additionally, the LIBRETTO-001 trial focused specifically on brain metastases originating from NSCLC, highlighting the efficacy of selpercatinib in shrinking metastases [43]. The overexpression of the *RET* gene is of functional importance, raising the possibility that *RET* inhibitors such as selpercatinib and pralsetinib could benefit BCBM patients with *RET* alterations, although this requires dedicated clinical studies.

#### HER3

HER3 is a protein receptor from the EGFR tyrosine kinase family, which is known to be expressed at higher levels in metastatic breast cancer in comparison to primary tumour samples [44]. HER3 has two ligands, namely heregulin (HRG) and neuregulin-1 (NRG1), with HRG being more commonly expressed in the brain [45]. A recent study using luminal breast cancer cell lines in vivo, highlighted the activity of HRG. HRG secretion in the brain promotes the dimerization of HER2/3 thus promoting the metastatic process [45]. 

Newer studies show that although the HER3 protein does not cause tumorigenesis directly, the HER2:HER3 heterodimer has significant ability to mediate HER-2 tumorigenesis in breast cancer [46]. This heterodimerization has become a key therapeutic target, with monoclonal antibodies developed to disrupt this process and potentially prevent the progression of BCBM.

There are currently 3 ongoing clinical trials on the use of HER3-Dxd, which is an antibody-drug conjugate. ICARUSBREAST02 (NCT06298084) investigates HER3-Dxd monotherapy and in combinations, in patients with advanced inoperable breast cancer [47]. NCT06172478 is ongoing and investigates HER3-Dxd monotherapy in advanced solid tumours, including breast cancer [48]. The two studies aforementioned had strict criteria regarding BCBM, with patients included only if they had clinically inactive BCBM.

Additionally, NCT04338747 is a phase IIa study of dendritic cell vaccines against HER2/HER3 and pembrolizumab in patients with asymptomatic brain metastases from TNBC or HER2 + BC or HR + BC [49]. This clinical trial may highlight the potential impact of immunotherapy against HER3 BCBM, the results of this may guide further clinical studies into targeted therapies for BCBM possessing HER3 mutations.

#### BCBM: Advances in Diagnostics

**Fig. 1:**
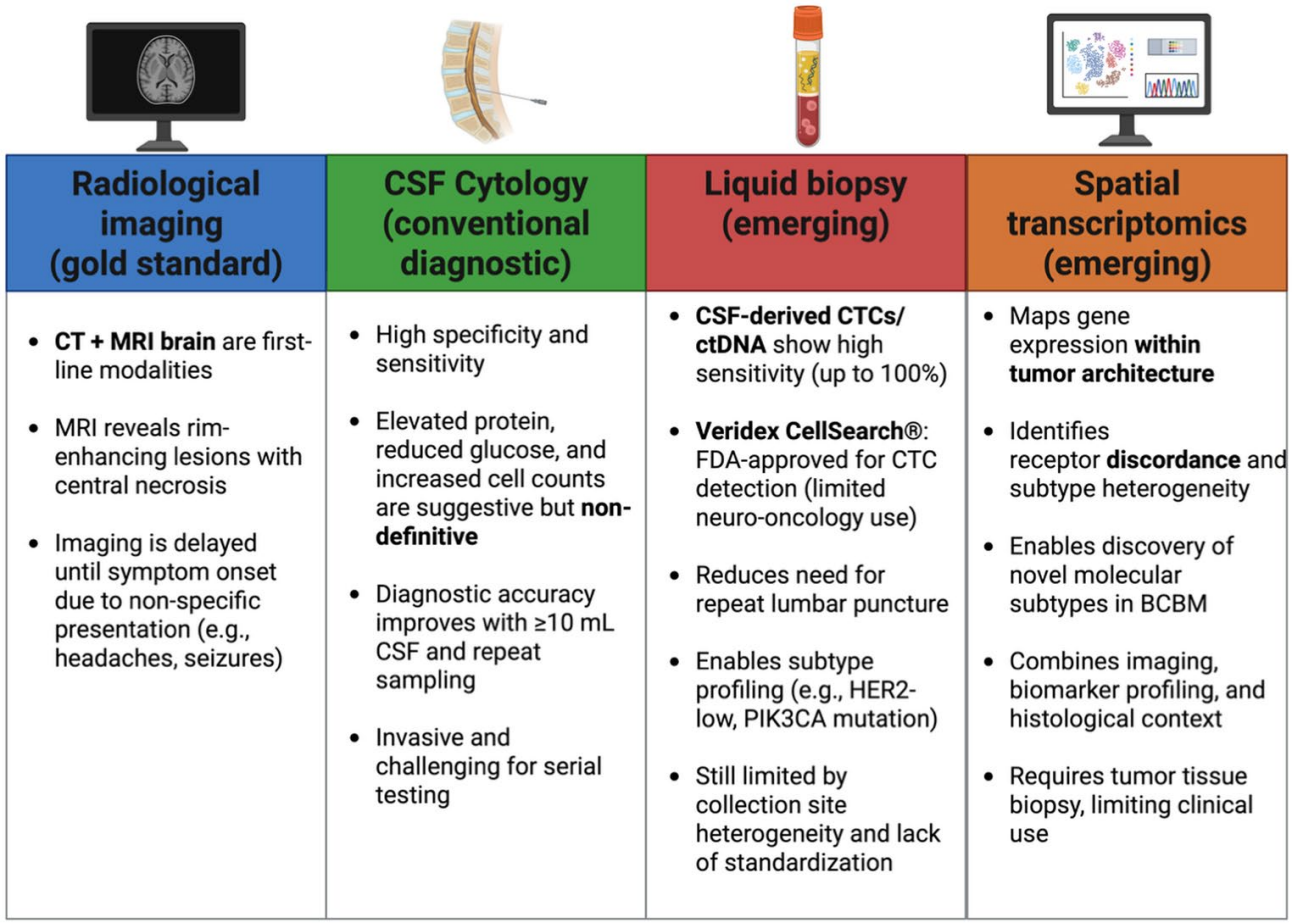
The figure gives an overview of current and emerging diagnostic approaches for brain metastases. Created from https://www.biorender.com/

The following section will provide a brief overview of the current ‘gold standard’ diagnostic methods by evaluating their efficacy, advantages, and limitations, followed by an exploration of emerging diagnostic approaches for BCBM (Fig. 1).

BCBM often presents with non-specific clinical symptoms, leading to a delay in diagnosis until the tumor burden is large enough to cause compressive effects. The diagnostic evaluation typically includes a comprehensive medical history, physical examination and neuroimaging. Additionally, cerebrospinal fluid (CSF) cytomorphological analysis, and, where necessary, tissue biopsy may be performed to assess changes in receptor status [2]. Commonly patients present with headaches, seizures, cognitive impairments, or focal neurological deficits [2]. CT followed by MRI brain are first-line imaging investigations in patients suspected to have BCBM [50]. MRI findings of rim-enhancing lesions with central non-enhancing regions are characteristic of the disease [51]. 

CSF abnormalities suggestive of metastatic brain or leptomeningeal disease, such as elevated cell counts, reduced glucose levels, and increased protein concentrations are not definitive markers [52]. CSF cytomorphological analysis, while highly specific for detecting solid tumours, suffers from low sensitivity due to the typically low volume of malignant cells in CSF. Older retrospective work reported a sensitivity of only 59% for a single CSF sample in patients later confirmed to have BCBM at autopsy, but this underestimates current diagnostic potential [53]. More recent studies have demonstrated substantially higher diagnostic accuracy with advanced approaches. For example, one prospective study by Darlix et al. showed 100% sensitivity and 77.3% specificity for circulating tumour cell (CTC) detection in CSF among patients with confirmed leptomeningeal or intraparenchymal metastatic breast cancer, aligning with previous reports of sensitivities of 78–100% and specificities of 84–100% [54, 55]. Le Rhun et al. successfully identified CTCs in 5 mL of CSF using the Veridex CellSearch^®^ system, which employs ferromagnetic EpCAM antibodies and remains the only FDA-approved methodology for enumerating CTCs in patients with breast, prostate, colorectal, or lung cancers [56, 57]. Importantly, CTC assays reduce the need for multiple CSF samples, addressing a major limitation of conventional cytology [58, 59]. Current guidelines still recommend analysing at least 10 mL of CSF and performing repeated sampling to optimize diagnostic accuracy [60, 61], but these advances suggest that liquid biopsy methods may help overcome some of the historic limitations of cytology. Despite these advantages, CSF collection is invasive and poses challenges for repeated sampling. Furthermore, the heterogeneity of CSF composition based on collection site (e.g., thoracic vs. lumbar spine) necessitates a standardized sampling protocol [62]. Longitudinal sampling at different diagnostic stages, during treatment, and in follow-up care could enable more comprehensive analyte monitoring and assessment of recurrence. However, further research is required to establish the optimal role of liquid biopsies in BCBM diagnostics, whether as a primary diagnostic tool, a surveillance strategy, or both.

Beyond CTCs, liquid biopsy approaches also include analysis of cell-free DNA (cfDNA) and circulating tumour DNA (ctDNA), which are under active investigation, with limited current clinical validation in BCBM [63]. While widely utilized in oncology for prognostication, treatment stratification, and post-therapy surveillance, their application in neuro-oncology remains limited [64]. 

A promising second avenue for future diagnostic advancements is the field of spatial transcriptomics. Despite significant progress, cancer treatment still encounters many challenges, including drug resistance, recurrence, and metastasis. High-throughput transcriptomics, commonly referred to as RNA sequencing, has enabled the identification of numerous alterations in gene expression across various tumor types [59]. However, traditional bulk sequencing methods lack the resolution necessary to capture details of the tumor microenvironment and cellular heterogeneity [65, 66]. Similarly, techniques such as single-cell RNA sequencing face limitations, including the dissociation of cells from their original spatial context within the tissue. Spatial transcriptomics can mitigate some of these limitations by combining biomarker analysis, imaging, genetic sequencing, and bioinformatics to map gene expression within intact tissue samples. This approach reveals the spatial distribution of diverse cell types within tissues and elucidates the interactions between these cell populations, providing a more comprehensive understanding of the tumor microenvironment and its complexity [62]. For example, a study utilizing spatial transcriptomics analyzed the genetic landscape of brain metastases originating from TNBC. The researchers compared the molecular characteristics of primary TNBC tumors with their corresponding brain metastases, uncovering distinct genetic profiles between the tumor cells and the surrounding tumor microenvironment. This comprehensive analysis provided insights into the molecular subtypes present in BCBM, highlighting the heterogeneity between primary and metastatic sites [67]. Specific application of spatial transcriptomics in BCBM is used in the identification of molecular subtypes of breast cancer, as frequently the metastatic lesion will differ from the primary cancer [68]. Not only does this allow for a better understanding of the specific tumor microenvironment, but high-resolution single-cell transcriptome sequencing, combined with spatial analysis, enables the visualization of cellular heterogeneity in both primary and metastatic breast cancer. This approach facilitates both the identification of metastasis-related cell subsets and also contributes to a better understanding of the metastatic process and improves diagnostic accuracy [69]. Furthermore, spatial transcriptomics has been applied to identify new breast cancer subtypes by analyzing the spatial distribution of gene expression within tumors. This approach provides insights into tumor heterogeneity and supports the development of individualized treatment strategies, specific to the metastatic subtype [68]. 

Spatial transcriptomics, however, does not come without its current limitations: the need for invasive testing, high cost, long turn-around times, and lack of standardization process to name a few [59, 70]. A comparison of these diagnostic tools is provided in Table 1.

**Table 1:** Comparison of diagnostic tools

	Sensitivity	Invasiveness	Spatial Resolution	Cost
CSF	High	High	N/A	Low
Liquid Biopsies	High	Low	N/A	Low
Spatial Transcriptomics	Undetermined, predominantly used as a research tool	Very high	High, varies with technique used	High

### Receptor Discordance between Primary Tumour and Brain Metastasis

Receptor discordance, defined as the variation in ER, progesterone receptor (PR), HER2 status between primary breast cancer and metastatic sites, is a well-documented phenomenon in metastatic breast cancer (MBC) [71–73]. This biological and clinical challenge is particularly relevant in BCBM, where receptor discordance between primary and metastatic sites remains a clinically significant issue. Although rates of discordance in BCBM are not consistently higher than those observed in other metastatic sites such as bone, changes in receptor status have been reported in a substantial proportion of cases, with one large multicentre study reporting an overall discordance rate of 36.3% and receptor-specific rates of 16.7% for ER, 25.2% for PR, and 10.4% for HER2 [74, 75]. These shifts can directly influence therapeutic strategies and highlight the broader importance of reassessing receptor status in BCBM.

Supporting this, two meta-analyses have highlighted significant discordance rates: ER discordance was observed in 19–20% of cases, PR discordance in 30–33%, and HER2 discordance in 8–10%. Notably, brain metastases exhibit an ER discordance rate of approximately 20.8%, reflecting a substantial shift in receptor expression profiles within the central nervous system (CNS) microenvironment, and subtype switching has been observed in nearly one-quarter of patients, often involving hormone receptor loss or HER2 gain [72, 75]. Importantly, 14.8% of patients who were HER2- at baseline developed HER2 + brain metastases, creating potential therapeutic opportunities, while receptor loss, particularly ER- has been associated with worse survival outcomes [75]. 

Several mechanisms contribute to receptor discordance in metastatic breast cancer, including technical variability in immunohistochemical (IHC) staining, tumour heterogeneity, and clonal evolution under therapeutic pressure. Bone and brain metastases, in particular, are susceptible to these alterations, likely due to distinct microenvironmental influences and selective pressures favouring specific tumour subpopulations.

- **Tumour Heterogeneity and Clonal Selection**: The existence of intra-tumour heterogeneity suggests that primary breast tumours comprise diverse cellular subpopulations with variable receptor expression. As metastases develop, certain clones may be preferentially selected, leading to a receptor status distinct from that of the primary tumour [73, 76–79].
- **Therapeutic Influence**: Systemic therapies, particularly endocrine and HER2-targeted treatments, may drive receptor conversion. Endocrine therapy, for instance, can induce loss of ER expression, rendering initially hormone receptor-positive (HR+) tumours hormone receptor-negative (HR−). Similarly, HER2-targeted agents such as trastuzumab can alter HER2 expression, affecting treatment responsiveness [80–85]. Emerging data also point to a distinct pattern of HER2-low discordance, wherein some metastatic lesions exhibit low-level HER2 expression despite a HER2- primary tumour. This phenomenon has important therapeutic relevance, particularly with the advent of novel agents targeting HER2-low disease [86].
- **Microenvironmental Adaptation**: The CNS presents a unique metastatic niche, characterized by the blood-brain barrier, differential immune surveillance, and metabolic constraints. These factors may drive adaptive changes in tumour cell receptor expression, promoting discordance [72, 75, 87, 88].

Prospective studies, including the DESTINY and BRITS trials, have assessed the impact of re-biopsy in metastatic breast cancer, revealing that treatment decisions were altered in 14.2% of patients based on receptor reassessment [89, 90]. In the context of BCBM specifically, these modifications can be particularly consequential, as receptor loss has been linked to inferior survival while HER2 acquisition may improve outcomes [75]. However, whether these modifications translate into improved overall survival remains debated, necessitating further investigation.

### Advancing Therapeutic Strategies in BCBM

#### Radiation Based Therapies

The treatment of brain metastases from breast cancer is often complex due to the varying presentations of the disease and the evolving array of treatment options. Traditional treatments like whole-brain radiotherapy (WBRT) have, to some extent, fallen out of favor with the rise of more targeted approaches such as stereotactic radiosurgery (SRS) and hippocampal-avoidant whole-brain radiotherapy (HA-WBRT). The ASCO-SNO-ASTRO guidelines recommend SRS for patients with 1–4 lesions and HA-WBRT for patients with a projected survival of 4 months or more who do not have hippocampal lesions [91]. Guidelines from EANO-ESMO align with these recommendations, advising WBRT primarily for patients with more than ten brain lesions, those with uncontrolled extra-CNS disease, or individuals with a life expectancy of less than three months [92]. For all other cases, treatment recommendations include surgery combined with SRS or stereotactic radiotherapy, surgery with pharmacotherapy, or pharmacotherapy alone, depending on the patient’s condition and tumor characteristics [92]. 

This progressive shift away from WBRT is not unfounded. WBRT often results in cognitive decline lasting 5–8 months and, in some cases, 9–15 months, with a greater risk of long-term effects. In contrast SRS has been shown to impair cognitive function, the effects are typically limited to 1–4 months, after which most patients return to baseline cognitive levels [93]. The reduced cognitive impact, combined with other therapeutic benefits, has been a significant factor driving the shift from WBRT.

WBRT has also been supplanted in certain circumstances by HA-WBRT. Using the Montreal Cognitive Assessment (MoCA) grading system, a randomized controlled study compared the cognitive function of patients receiving WBRT to those receiving HA-WBRT for brain metastases from breast and lung cancers [94]. The results showed significantly higher MoCA scores in the HA-WBRT group [94]. The use of HA-WBRT is expected to grow, as it offers many of the same benefits as WBRT but with fewer side effects. Ongoing trials are further investigating its value in treating brain metastases (NCT03075072) [95]. 

Despite these advancements, WBRT retains a niche role. It remains valuable for managing microscopic tumours, preventing recurrences, and enhancing the effects of other treatments, such as surgery. A study comparing WBRT combined with SRS to SRS alone found significantly lower recurrence rates and improved overall survival in the combined treatment group [96]. DERGO guidelines also recommend WBRT as an option for patients with 5–10 brain metastases (with a cumulative volume of less than 15 mL) and for those with a limited number of brain metastases (5–10 lesions, cumulative volume < 15 mL) following resection. Additionally, WBRT is advised for patients with multiple symptomatic lesions when hippocampal avoidance is not feasible, demonstrating WBRT’s maintained relevancy in treatment [97]. 

### Systemic Therapies with Clinical Evidence in BCBM

Alongside advancements in radiation techniques, combinations between various systemic therapies and radiation therapies are being explored to optimize patient outcomes. A study assessed irinotecan and temozolomide in 30 patients, nearly all of whom had brain metastases from various breast cancer subtypes. Most of these patients had previously undergone SRS or WBRT and had experienced disease progression. Following the initiation of the drug regimen, only one patient showed further disease progression, and effectiveness was observed across all cancer subtypes [98]. This demonstrates the proven clinical benefit of combining systemic therapies with radiotherapy in BCBM patients.

#### HER2 + Disease

Treatments targeting HER2 + brain metastases have also been progressing. Tucatinib, for example, was recently studied in combination with trastuzumab and capecitabine. In a trial involving 291 patients with brain metastases, the tucatinib group demonstrated significantly improved survival outcomes (21.6 months versus 12.5 months in the control group treated with placebo, trastuzumab, and capecitabine) [99]. The National Comprehensive Cancer Network guidelines also list this as a preferred treatment regimen for patients previously treated with anti-HER2 drugs [100]. Thus, tucatinib remains a valuable option for difficult-to-target metastases and an important tool for combination therapy.

Trastuzumab-deruxtecan has also been shown to be a highly effective treatment for HER2 + brain metastases. In a population of 15 patients, the drug had a response rate of 73.3% with 13.3% of patients experiencing complete responses and 60% of patients. Additionally, treatment was overall well tolerated, and quality of life was not impaired [101]. These findings suggest that trastuzumab-deruxtecan offers a promising therapeutic option for patients with HER2‑positive BCBM, potentially improving outcomes in this difficult-to-treat population. Furthermore, DESTINY-Breast12, a large prospective clinical trial demonstrated trastuzumab-deruxtecan to have a 12 month progression-free survival of 61.6% for HER2 + with BCBM and low discontinuation rates from adverse events [102]. Further exemplifying the potency of Trastuzumab Deruxtecan, the recent DESTINY-Breast09 Trial demonstrated that Trastuzumab Deruxtecan plus Pertuzumab for HER2 + metastatic breast cancer was superior to the standard first-line therapy of a trastuzumab, pertuzumab, and a taxane (median progression-free of 40.7 vs. 26.9 months) [103]. Although the trial did not explicitly examine intracranial efficacy, they found that progression-free survival benefit was consistent across BCBM patients and state future intent to evaluate intracranial efficacy [103]. Despite the proven potency of trastuzumab, it and other drugs can be improving their ability to penetrate the BBB [104]. To address this challenge, studies are attempting strategies to enhance penetration, including combining trastuzumab with other treatments such as IA NEO100, an agent designed to increase BBB permeability [104]. IA NEO aims to enhance CNS activity reversibly opening the BBB, though further research is needed to assess its use in a clinical environment [104]. 

Other FDA approved agents for HER2 + BCBM include capecitabine in combination with trastuzumab and tucatinib [105]. This combination is approved for patients with unresectable or metastatic HER2 + disease after failing one line of anti HER2 regimens [105]. This approval was based on the HER2CLIMB trial which found this drug combination to have a progression-free survival of 24.9% at one year compared to 0% of the placebo group in brain metastasis patients [106]. Capecitabine has demonstrated CNS efficacy in the past making it a valuable treatment for BCBM [107]. Capecitabine has also been studied in combination with the drug neratinib compared with lapatinib plus capecitabine in the randomized phase III NALA trial in previously treated HER2 + metastatic breast cancer patients [108]. Subgroup analysis from this trial on BCBM patients revealed neratinib plus capecitabine had a better progression-free survival at 24 months compared to lapatinib plus capecitabine with a mean of 7.8 vs. 5.5 months (HR 0.66, 95% CI 0.41–1.05) [109]. These findings suggest strong CNS efficacy of neratinib along with capecitabine. Another potent treatment for HER2 + BCBM with proven CNS efficacy is trastuzumab emtansine (T-DM1). The KAMILLA trial, a phase IIIb multicenter study evaluated T-DM1 in HER2 + patients with unresponsive advanced or metastatic breast cancer [110]. Subgroup analysis of BCBM patients revealed in 126 patients with measurable BCBM, 42.9% (95% CI 34.1–52.0) achieved a ≥ 30% reduction in lesion size. Ongoing trials such as the TUCATEMEB trial (NCT05673928) are further evaluating tucatinib with T-DM1 in treating HER2 + BCBM as well as other cancers [111]. T-DM1 adds to the multitude of available drugs with CNS efficacy in patients with HER2 + BCBM [110, 112]. 

#### ER + Disease

In comparison to HER2+, treatment options for ER + BCBM have been limited. Many anti hormonal drugs have been studied though they face significant challenges as not all of them can cross the BBB [113]. Novel alternative such as selective estrogen receptor degraders (SERDs) including elacestrant may have greater ability to cross the BBB [114]. Elacestrant has been approved in 2023 for advanced metastatic breast cancer in HER2-, ER+, ESR1-mutated patients with resistant disease [115]. This came after multiple phase 1 clinical trials (NCT02650817, NCT02338349) studying elacestrant for advanced breast cancer found antitumor activity in heavily pretreated ER + breast cancer [116, 117]. Despite this, two phase 1 studies in postmenopausal women found low elacestrant concentrations in CSF suggesting the need for more human studies to confirm its efficacy in BCBM patients [114]. 

Further expanding the use of novel SERDs imlunestrant is another drug of the same class with proven efficacy for ER+, ESR1-mutated patients with advanced breast cancer. In the EMBER-3 trial (NCT04975308), which enrolled 874 with ER-positive HER2- treatment resistant advanced breast cancer, imlunestrant demonstrated significantly greater progression-free survival compared to standard endocrine monotherapy, which consisted of oral exemestane or intramuscular fulvestrant for patients with *ESR1* mutations (mean survival time, 2.6 months; 95% CI, 1.2 to 6.9; *P* < 0.001) [118]. The significance of this benefit did not extend to the overall cohort, representing a limitation in the broader usage of imlunestrant [118]. 

Imlunestrant also may help address the limitations of treating ER + BCBM due to its ability as a CNS penetrant. In the EMBER-3 trial, there was significantly lower incidence of CNS progression in patients receiving imlunestrant compared to standard therapy in the ESR1 mutated subgroup (1.5% vs. 6.7%, cause-specific hazard ratio, 0.18; 95% CI, 0.04 to 0.90). However, this benefit was not significant in the overall population, and the trial excluded patients with symptomatic or untreated BCBM indicating a greater need for clinical data to determine its efficacy in treating BCBM [118]. 

### Investigational Therapies in BCBM

Studies have explored combining radiotherapy with targeted treatments such as poly-ADP ribose polymerase (PARP) inhibitors like veliparib. A study tested veliparib in a group of 25 breast cancer brain metastasis patients undergoing WBRT and demonstrated a partial or complete response in 41% of patients [119]. However, veliparib’s original results were disappointing, as veliparib failed to significantly improve pathological complete response when added to treatment regimens compared to control group in treatment for TNBC [120]. Nevertheless, some studies remain optimistic, exploring its effectiveness in various combination therapies and pathologies.

Beyond veliparib, other PARPis have shown more promising outcomes. PARPis such as olaparib and talazoparib have been approved for the treatment of metastatic breast cancer in BRCA1 and BRCA2 carriers and have recently demonstrated efficacy in patients with PALB2 mutations [121]. Because PARPis are used in both triple negative and hormone receptor positive breast cancers with germline *BRCA1* or *BRCA2* mutations, their continued development is particularly valuable. Moreover, PARPis such as olaparib have shown relevant therapeutic levels in the brain, demonstrating promise in penetrating the BBB, a challenge encountered by many therapeutic agents in treating BCBM [122]. Despite this, more clinical evidence is needed to confirm the efficacy of drugs such as olaparib in the brain.

Immunotherapies have increasingly emerged as versatile tools for managing BCBM. Evidence of this can be seen in newly developed drugs targeting programmed death-ligand 1 (PD-L1), including atezolizumab. This drug was tested in a combination therapy study with pertuzumab and trastuzumab for HER2 + breast cancer (NCT03417544) [123, 124]. The clinical benefit rate at 18 weeks was 42.1%, with only 2 out of 19 patients achieving a partial response. PD-L1 inhibitors are still being studied as numerous ongoing studies are investigating its use (NCT06418594) [125]. PD-L1 inhibitors are also used for treatment in TNBC making it potentially an effective therapy for various subtypes of brain metastasis in the future. However, due to the PD-L1 inhibitor’s large molecular size, there are concerns that this drug class may not effectively penetrate the BBB, and further research is warranted for clarification [126]. In parallel, a recent study in mice, portrayed the way in which brain astrocytes can induce overexpression of CDK5 in BCBM, thus encouraging and allowing for outgrowth [127]. CDK5 can suppress MHC-I expression on cancer cells, preventing T-cells from recognising BCBM allowing for the evasion of the immune response, however, studies are needed to evaluate their use clinically [127]. Another novel therapeutic agent, roscovitine, used independently or alongside immune checkpoint inhibitors, showed a significantly reduced BCBM burden, with increased levels of tumour infiltration by T-cells in mice [127]. This highlights the role of CDK5 in immune evasion, as well as the potential for CDK5 inhibitors to be used as a means of restoring immune recognition, as well as improved tumour control [127]. Nonetheless, more evidence is required to evaluate their clinical efficacy.

Another promising drug class is CDK12 inhibitors. CDK12 has been implicated in resistance to anti-HER2 treatments [128]. Several ongoing trials are evaluating the effectiveness of drugs like CT7439 in advanced breast cancer (NCT06600789), offering hope for improved prognoses in cases of resistant metastases [129]. A recent study found that CDK12 was the most frequently rearranged gene in brain metastases from breast cancer, with a significantly higher rearrangement rate in HER2 + cases compared to HER2- ones [130]. This suggests that CDK12 not only plays a critical role in the pathogenesis of BCBM but may also contribute to resistance against anti-HER2 therapies, but more research is needed to assess its clinical utility.

### Preclinical and Functional Precision Medicine Approaches

An interesting study of 12 BCBM and their respective primary tumour samples, were used to identify potential treatment targets [131]. Six novel patient-derived xenografts (PDXs) were used as drug screening platforms. The PDXs were treated with a panel of more than 350 drugs [131]. Alterations in the PI3K pathway were reported to be the most predictive for drug efficacy in PDXs [131]. The highest sensitivity was demonstrated to histone deacetylase (HDAC) inhibitors and proteasome inhibitors [131]. It is important to note, that these drugs would not have been identified as they are not on routine NGS sequencing panels, functional drug screening is very important in this individualized approach [131]. This study highlighted the importance of using accurate preclinical models such as PDXs, and the importance of functional drug response assessments in a wide range of targets, to allow for the identification of effective therapies to optimize treatment options for BCBM patients. These xenografts provide valuable preclinical evidence for emerging drug classes and may pave the way for personalized BCBM treatment, although clinical validation is still required. Details of the aforementioned pharmacologic trials are summarized in Table 2.

**Table 2:** Overview of pharmacologic trials evaluated for BCBM

Study Title (NCT)	Drug Interventions	Phase(s)	BCBM-Specific Findings	Reference(s)
Phase II study of irinotecan and temozolomide in breast cancer patients with progressing central nervous system disease (NCT00617539)	Irinotecan hydrochloride Temozolomide	Phase II	Two patients achieved confirmed partial responses in the CNS, while five maintained stable disease for at least 16 weeks.	[98]
Tucatinib vs Placebo, Both in Combination With Trastuzumab and Capecitabine, for Previously Treated ERBB2 (HER2)-Positive Metastatic Breast Cancer in Patients With Brain Metastases: Updated Exploratory Analysis of the HER2CLIMB Randomized Clinical Trial (NCT02614794)	Tucatinib Capecitabine Trastuzumab Placebo	Phase II	Tucatinib, when used alongside trastuzumab and capecitabine, extended median overall survival by 9.1 months in patients with brain metastases and lowered the likelihood of new brain lesions or death as the first event by 45.1% across all participants.	[99]
Trastuzumab deruxtecan in HER2-positive breast cancer with brain metastases: a single-arm, phase 2 trial (NCT04752059)	Trastuzumab deruxtecan	Phase II	Trastuzumab deruxtecan achieved its primary goal, demonstrating a 73.7% intracranial response rate.	[132]
A Phase II Study of Atezolizumab, Pertuzumab, and High-Dose Trastuzumab for Central Nervous System Metastases in Patients with HER2-Positive Breast Cancer (NCT03417544)	Atezolizumab Pertuzumab Trastuzumab	Phase II	Out of 19 participants, two experienced a confirmed partial response in the brain, resulting in a CNS overall response rate of 10.5% (90% CI: 1.9%–29.6%). As the trial did not meet its predefined efficacy criteria, it was discontinued early.	[123]
A Study of T-DXd in Participants With or Without Brain Metastasis Who Have Previously Treated Advanced or Metastatic HER2 Positive Breast Cancer (NCT04739761)	Trastuzumab deruxtecan	Phase III	Trastuzumab deruxtecan achieved a CNS progression-free survival of 58.9% demonstrating high intracranial activity.	[102]
Trastuzumab Deruxtecan plus Pertuzumab for HER2-Positive Metastatic Breast Cancer (NCT04784715)	Trastuzumab deruxtecan Placebo Taxane Pertuzumab Trastuzumab	Phase III	Trastuzumab deruxtecan plus pertuzumab improved first-line progression-free survival (40.7 vs 26.9 months) compared with the standard regimen of taxane, trastuzumab, and pertuzumab in HER2-positive metastatic breast cancer. This benefit was also seen in patients with BCBM.	[103]
A Study of Tucatinib vs. Placebo in Combination With Capecitabine & Trastuzumab in Patients With Advanced HER2+ Breast Cancer (NCT02614794)	Tucatinib Capecitabine Trastuzumab Placebo	Phase II	Patients with BCBM had a 24.9% progression free survival with the tucatinib combination vs 0% with the placebo combination (HR 0.48; 95% CI 0.34–0.69; P<0.001).	[106]
Imlunestrant with or without Abemaciclib in Advanced Breast Cancer (NCT04975308)	Imlunestrant Exemestane Fulvestrant Abemaciclib	Phase III	Cumulative incidence of CNS progression was significantly lower in the imlunestrant group compared to standard treatment (1.5% vs 6.7%; hazard ratio 0.18, 95% CI 0.04–0.90 in ESR1-mutated). However, this benefit was only significant in the ESR1-mutated subgroup. (1.6% vs 3%; hazard ratio, 0.47; 95% CI, 0.16 to 1.38 in overall population)	[118]
A Study of Neratinib Plus Capecitabine Versus Lapatinib Plus Capecitabine in Patients With HER2+ Metastatic Breast Cancer Who Have Received Two or More Prior HER2 Directed Regimens in the Metastatic Setting (NCT01808573)	Neratinib Capecitabine Lapatinib	Phase III	In 101 patients with CNS metastases, median PFS was 7.8 months with neratinib with capecitabine vs 5.5 months with lapatinib with capecitabine; intracranial objective response rate was 26.3% vs 15.4%, respectively.	[108, 109]
A Study of Trastuzumab Emtansine in Participants With Human Epidermal Growth Factor Receptor 2 (HER2)-Positive Breast Cancer Who Have Received Prior Anti-HER2 And Chemotherapy-based Treatment (NCT01702571)	Trastuzumab Emtansine	Phase III	Among all patients with BCBM (n = 398), median PFS was 5.5 months (95% CI 5.3–5.6) and median OS was 18.9 months (95% CI 17.1–21.3). In those with measurable BCBM (n=126), 42.9% (95% CI 34.1–52.0) achieved a ≥30% reduction in lesion size.	[110, 112]
A Phase II Study of Tucatinib and Ado-trastuzumab Emtansine (T-DM1) in Patients With HER2-positive Metastatic Solid Tumours and Metastases to Brain (NCT05673928)	Trastuzumab emtansine Tucatinib	Phase II	Recruiting	[111]
Adebrelimab Plus Apatinib and Etoposide for the Treatment of HER2-Negative Breast Cancer Brain Metastasis (NCT06418594)	Adebrelimab Apatinib Etoposide	Phase II	Recruiting	[125]
A Modular Phase 1/2 Study with CT7439 in Participants with Solid Malignancies (including advanced breast cancer) (NCT06600789)	CT7439	Phase I/II	Recruiting	[129]

### Future Directions

Future directions in BCBM research increasingly incorporate developments in computational science and cancer neuroscience. These approaches are being explored to deepen understanding of disease mechanisms and improve clinical decision making.

### Machine Learning

Machine learning (ML) is garnering increasing attention for its potential to improve prognostication, treatment planning, and diagnosis in patients with BCBMs.

Initial ML applications as prognostic measures in BCBM involved the generation of models to predict survival. Li et al. utilized the clinical features of BCBM patients, from the Surveillance, Epidemiology, and End Results (SEER) database (2010–2019), to generate an XGBoost model capable of predicting survival. Impressive results were observed, particularly in forecasting 3-year survival on external datasets with an AUC of 0.936 [133]. This model allowed for further identification of underlying trends such as the added survival benefit of surgical intervention in HER2 + and HR-/HER2- subtypes of patients with BCBM and lack of benefit in the HR+/HER2- subtype [133]. 

More advanced applications in prognostication have included the utilization of AI to predict breast cancers with metastatic potential. The value of AI in this context has recently been demonstrated by Tan et al., in the development of an AI model utilizing clinical blood markers and ultrasound data to predict distant metastasis in patients with breast cancer [134]. Although this LightGBM model did not include analysis on brain metastases, the results of the combined model on external datasets were encouraging with an AUC of 0.918 [134]. In addition, the analysis of features utilized by the model allows further insight into biochemistry markers previously queried for their role in metastatic cancer. Such is seen in the exploration of CK-MB and CA153, which were highlighted as key features of their model, substantiating previous studies into their prognostic value [134]. A similar model was developed by Shiner et al., which encompassed brain metastases and displayed prominent levels of accuracy [135]. 

ML has been employed to detect BCBMs on imaging to automate and enhance diagnostics. Grovik et al. utilized a deep learning algorithm based on a convolutional neural network to analyze multisequence MRIs and effectively detect the presence of metastases as well as segment individual growths [136]. Similarly, Ziyaee produced a deep-learning model with high positive predictive value in delineating metastatic margins on MRI, a key aspect of treatment planning [137]. Interestingly, 9% of false-positives in this population were in fact true-positives missed on initial review and labelling, which highlights the power of this model to enhance early detection [137]. 

The field of radiomics is an emerging application of advanced ML capabilities in radiology. Although perfusion and permeability imaging have taken strides towards quantitative imagining, radiomics accesses pixel-level relationships and image features such as texture and morphology to draw conclusions [138]. Nowakowski et al. have summarized the diverse array of radiomic applications specific to brain metastases, including the prediction of response to stereotactic radiosurgery, differentiating radiation necrosis from local recurrence/progression, predicting mutation status, differentiating between primary and metastatic tumors, and lastly predicting the primary cancer site in the case of metastasis [139]. The breadth of information delivered by radiomic models may function as a form of virtual biopsy in research settings, with the potential to provide non-invasive support for patient stratification, treatment planning and detection of coexisting pathology in the future.

The application of ML in neuro-oncology holds immense promise, yet this enthusiasm must be tempered by a recognition of its current limitations. The NEVER study, which assessed publication bias in radiomics research, identified several key weaknesses that contribute to the growing disconnect between ML studies and their clinical translation [140]. Among these are the predominance of retrospective study designs (95%), reliance on private datasets (91%), single-institution settings (75%), and a lack of external validation (81%) [140]. With regards to BCBMs specifically, there is a particular scarcity of prospective literature, although integration of ML into brain metastasis clinical workflows has been initiated. Such an example can be seen in VBrain, an FDA-cleared deep learning model constructed for the diagnosis and segmentation of metastatic brain tumours [141]. Furthermore, many ML studies fail to compare their models to established diagnostic standards, making it difficult to assess their real-world utility [140]. Variability in ML methodologies further undermines reproducibility and raises concerns about the generalizability of these models across diverse populations [140]. A practical roadmap to overcome these challenges could include the utilization of common data elements (CDEs), as well as the establishment of multi-centre validation consortia. Past reviews have demonstrated how variations in segmentation as well as the application of external validation can augment the diagnostic accuracy of radiomic models [142]. CDEs may be applied in this context to not only standardize feature definitions and formats, but to harmonize segmentation protocols to ensure reproducibility. Shared validation data across institutions should afford models a wealth of heterogenous data to mimic a real-world landscape and improve generalizability. Establishing external validation as a consistent requirement is essential to narrowing the gap between academic literature and clinical practice. Such efforts could lay the groundwork for the prospective use of ML models and accelerate their integration into precision medicine.

### Cancer Neuroscience in BCBM

Breast cancer cells that enter the cerebral circulation must first engage and remodel the blood brain barrier. They influence endothelial cells, pericytes, and astrocytic end feet to loosen tight junctions and allow their arrest and passage into the parenchyma. Enzymes and transporters such as ENPP1 and molecular pathways that regulate endothelial permeability have been identified as key mediators of this early step [143, 144]. Once within the brain tissue, metastatic cells must survive a highly specialized environment. They adapt by exploiting local trophic signals and by adopting features of neuronal physiology, a process often described as neuronal mimicry. Evidence from patient samples and preclinical models demonstrates that these cells can utilize gamma aminobutyric acid as a metabolic substrate, expressing the associated transporters and enzymes, and in some cases forming pseudo synaptic contacts with neurons that allow access to neurotransmitter related nutrients [145]. Similar interactions have been observed with glutamatergic neurons, where tumour cells display sensitivity to N methyl D aspartate (NMDA) receptor signalling and may exploit neuronal firing to enhance their own proliferation [146]. 

Astrocytes emerge as critical partners in this process. Direct physical connections such as gap junctions allow the exchange of small molecules and microRNAs that reprogram astrocytes toward a tumour supportive phenotype, increasing the resistance of cancer cells to stress and therapy. Astrocyte derived signals can also activate cyclin dependent kinase 5 in tumour cells, promoting invasion [147]. This supportive microenvironment is further shaped by microglia, which respond to disseminated tumour cells with a range of programs. Although they can mount antitumor responses characterized by antigen presentation and pro inflammatory activity, microglia also release cytokines such as interleukin six and C-C motif chemokine ligand two that enhance tumour cell survival and motility [148]. Reprogramming microglia toward a sustained antitumor phenotype in experimental systems suppresses metastatic growth, highlighting their therapeutic potential.

Communication between tumour and brain cells is not limited to direct contact. Breast cancer cells with a propensity for brain colonization release extracellular vesicles carrying microRNAs that alter neuronal and astrocytic metabolism. For example, miR 199b 5p targets solute carrier transporters, leading to glutamate accumulation in the extracellular space, which in turn fuels tumour growth [149]. Other microRNAs have been linked to disruption of barrier integrity and modulation of the immune and glial landscape, suggesting their role as both biomarkers and therapeutic targets.

The interplay between tumour biology and the neural microenvironment varies by breast cancer subtype. HER2 + and triple negative disease have the highest rates of brain involvement, and their respective tumour cells exhibit distinct strategies for barrier interaction, neuronal mimicry, and reliance on glial support [144]. These biological differences influence drug penetration and treatment response, reinforcing the need for approaches that target neuron like signalling and glial contributions in addition to conventional local and systemic therapies.

Emerging work now connects barrier remodelling, neural circuit activity, astrocyte and microglia state changes, and vesicle mediated communication into a unified model of metastatic growth in the brain [143–149]. Future research priorities include mapping the specific neural circuits that promote tumour expansion in each subtype, charting the dynamic states of astrocytes and microglia in patients, and developing interventions that interrupt synaptic like signalling or restore the protective functions of the barrier and resident immune cells. Such advances may provide the mechanistic basis for therapies that complement current surgery, radiotherapy, and systemic treatment to more effectively control brain metastases in breast cancer.

### Limitations

This paper is a literature review, which brings several important limitations. A review depends on the quality and consistency of the published work it draws from. Many of the available studies on BCBM are small, retrospective, or based on experimental laboratory models. This means the findings may not fully reflect outcomes in broader patient groups. Some of the biological pathways and diagnostic methods discussed have only been tested in early research settings and are not yet supported by large clinical studies. In addition, new tools such as liquid biopsy, spatial transcriptomics, radiomics and machine learning vary widely in their methods and have limited evaluation in real world clinical care. Research on receptor changes in brain metastases is also limited because brain tissue samples are difficult to obtain. These factors mean that the conclusions of this review should be interpreted with caution. More large and carefully designed clinical studies are needed to confirm how these emerging approaches can be used in the management of BCBM.

## Conclusion

Future progress in BCBM will likely involve a coordinated approach that unites advances in computational science with deeper exploration of cancer neuroscience. Priorities include the standardization of spatial transcriptomics protocols specific to this disease to allow reproducible mapping of tumour-microenvironment interactions, the creation of multi-institutional consortia to externally validate radiomic and other ML models for clinical applicability, and the launch of prospective trials to determine the survival impact of receptor reassessment. Equally important is the continued investigation of neural circuit involvement, glial cell dynamics, and vesicle-mediated signalling as therapeutic targets, supported by functional drug screening in patient-derived models. Achieving these aims may help translate molecular, imaging, and neurobiological insights into precision strategies that can improve outcomes in this challenging patient population.

## Key References

- Jagust P, Powell AM, Ola M, Watson L, de Pablos-Aragoneses A, García-Gómez P, et al. RET overexpression leads to increased brain metastatic competency in luminal breast cancer. J Natl Cancer Inst. 2024;116(10):1632-44.
  - ○ Identified RET overexpression as a driver of oestrogen receptor positive breast cancer growth in the brain, supporting RET inhibition as a potential treatment.
- Hulsbergen AFC, Claes A, Kavouridis VK, Ansaripour A, Nogarede C, Hughes ME, et al. Subtype switching in breast cancer brain metastases: a multicenter analysis. Neuro Oncol. 2020;22(8):1173-81.
  - ○ It provides essential insight by demonstrating subtype switching and receptor discordance in breast cancer brain metastases, directly informing prognosis and highlighting the need for receptor reassessment to optimize treatment decisions.
- Vogelbaum MA, Brown PD, Messersmith H, Brastianos PK, Burri S, Cahill D, et al. Treatment for brain metastases: ASCO-SNO-ASTRO guideline. Oxford University Press US; 2022.
  - ○ This guideline provides evidence-based recommendations for the treatment of brain metastases, offering clear guidance on surgery, radiosurgery, and radiation therapy in different clinical scenarios.
- Lin NU, Murthy RK, Abramson V, Anders C, Bachelot T, Bedard PL, et al. Tucatinib vs placebo, both in combination with trastuzumab and capecitabine, for previously treated ERBB2 (HER2)-positive metastatic breast cancer in patients with brain metastases: updated exploratory analysis of the HER2CLIMB randomized clinical trial. JAMA oncology. 2023;9(2):197-205.
  - ○ This study provides strong clinical evidence that tucatinib can extend survival in patients with HER2 positive metastatic breast cancer and brain metastases, addressing a group with limited effective treatment options.
- Bartsch R, Berghoff AS, Furtner J, Marhold M, Bergen ES, Roider-Schur S, et al. Final outcome analysis from the phase II TUXEDO-1 trial of trastuzumab-deruxtecan in HER2-positive breast cancer patients with active brain metastases. Neuro-oncology. 2024;26(12):2305-15.
  - ○ This study demonstrates that trastuzumab deruxtecan provides durable disease control in patients with active HER2 positive breast cancer brain metastases, supporting its role as an effective treatment option.

## Data Availability

No datasets were generated or analysed during the current study.

## References

[1] Darlix A, Louvel G, Fraisse J, Jacot W, Brain E, Debled M, et al. Impact of breast cancer molecular subtypes on the incidence, kinetics and prognosis of central nervous system metastases in a large multicentre real-life cohort. Br J Cancer. 2019;121(12):991–1000. 31719684 10.1038/s41416-019-0619-y PMC6964671

[2] Raghavendra AS, Ibrahim NK. Breast cancer brain metastasis: a comprehensive review. JCO Oncol Pract. 2024. 10.1200/op.23.00794. 38748968 10.1200/OP.23.00794 PMC11477856

[3] Liddelow SA, Barres BA. Reactive astrocytes: production, function, and therapeutic potential. Immunity. 2017;46(6):957–67. 28636962 10.1016/j.immuni.2017.06.006

[4] Priego N, Zhu L, Monteiro C, Mulders M, Wasilewski D, Bindeman W, et al. STAT3 labels a subpopulation of reactive astrocytes required for brain metastasis. Nat Med. 2018;24(7):1024–35. 29892069 10.1038/s41591-018-0044-4

[5] Hosonaga M, Saya H, Arima Y. Molecular and cellular mechanisms underlying brain metastasis of breast cancer. Cancer Metastasis Rev. 2020;39(3):711–20. 32399646 10.1007/s10555-020-09881-y PMC7497304

[6] Daly GR, Naidoo S, Alabdulrahman M, McGrath J, Dowling GP, AlRawashdeh MM, et al. Screening and testing for homologous recombination repair deficiency (HRD) in breast cancer: an overview of the current global landscape. Curr Oncol Rep. 2024;26(8):890–903. 38822929 10.1007/s11912-024-01560-3 PMC11300621

[7] Easton DF, Pharoah PD, Antoniou AC, Tischkowitz M, Tavtigian SV, Nathanson KL, et al. Gene-panel sequencing and the prediction of breast-cancer risk. N Engl J Med. 2015;372(23):2243–57. 26014596 10.1056/NEJMsr1501341 PMC4610139

[8] Daly GR, AlRawashdeh MM, McGrath J, Dowling GP, Cox L, Naidoo S, et al. PARP inhibitors in breast cancer: a short communication. Curr Oncol Rep. 2024;26(2):103–13. 38236558 10.1007/s11912-023-01488-0 PMC10891270

[9] Galland L, Ballot E, Mananet H, Boidot R, Lecuelle J, Albuisson J, et al. Efficacy of platinum-based chemotherapy in metastatic breast cancer and HRD biomarkers: utility of exome sequencing. NPJ Breast Cancer. 2022;8(1):28. 35246547 10.1038/s41523-022-00395-0 PMC8897409

[10] André F, Ciruelos EM, Juric D, Loibl S, Campone M, Mayer IA, et al. Alpelisib plus fulvestrant for PIK3CA-mutated, hormone receptor-positive, human epidermal growth factor receptor-2-negative advanced breast cancer: final overall survival results from SOLAR-1. Ann Oncol. 2021;32(2):208–17. 33246021 10.1016/j.annonc.2020.11.011

[11] Carter M, Yome J, Marcil M, Martin C, Vanhorne J, Mulligan L. Conservation of RET proto-oncogene splicing variants and implications for RET isoform function. Cytogenet Cell Genet. 2001;95(3–4):169–76. 12063395 10.1159/000059341

[12] Knijnenburg TA, Wang L, Zimmermann MT, Chambwe N, Gao GF, Cherniack AD, et al. Genomic and molecular landscape of DNA damage repair deficiency across the cancer genome atlas. Cell Rep. 2018;23(1):239–54. e6. 29617664 10.1016/j.celrep.2018.03.076 PMC5961503

[13] Habaka M, Daly GR, Shinyanbola D, Alabdulrahman M, McGrath J, Dowling GP, et al. PARP inhibitors in the neoadjuvant Setting; A comprehensive overview of the rationale for their Use, past and ongoing clinical trials. Curr Oncol Rep. 2025;27(5):533–51. 40192976 10.1007/s11912-025-01669-z PMC12081494

[14] Robson ME, Im S-A, Senkus E, Xu B, Domchek SM, Masuda N, et al. OlympiAD extended follow-up for overall survival and safety: olaparib versus chemotherapy treatment of physician’s choice in patients with a germline BRCA mutation and HER2-negative metastatic breast cancer. Eur J Cancer. 2023;184:39–47. 36893711 10.1016/j.ejca.2023.01.031 PMC10585240

[15] Litton JK, Hurvitz SA, Mina LA, Rugo HS, Lee KH, Gonçalves A, et al. Talazoparib versus chemotherapy in patients with germline BRCA1/2-mutated HER2-negative advanced breast cancer: final overall survival results from the EMBRACA trial. Ann Oncol. 2020;31(11):1526–35. 32828825 10.1016/j.annonc.2020.08.2098 PMC10649377

[16] Staniszewska AD, Pilger D, Gill SJ, Jamal K, Bohin N, Guzzetti S, et al. Preclinical characterization of AZD9574, a Blood-Brain barrier penetrant inhibitor of PARP1. Clin Cancer Res. 2024;30(7):1338–51. 37967136 10.1158/1078-0432.CCR-23-2094

[17] Xiong Y, Guo Y, Liu Y, Wang H, Gong W, Liu Y, et al. Pamiparib is a potent and selective PARP inhibitor with unique potential for the treatment of brain tumor. Neoplasia. 2020;22(9):431–40. 32652442 10.1016/j.neo.2020.06.009 PMC7350150

[18] A Phase 1 Study of MOMA-313 Given as Monotherapy or in Combination With a PARP Inhibitor in Participants With Advanced or Metastatic Solid Tumors [Internet]. 2024. Available from: https://clinicaltrials.gov/study/NCT06545942

[19] Smith HL, Willmore E, Prendergast L, Curtin NJ. ATR, CHK1 and WEE1 inhibitors cause homologous recombination repair deficiency to induce synthetic lethality with PARP inhibitors. Br J Cancer. 2024;131(5):905–17. 38965423 10.1038/s41416-024-02745-0 PMC11369084

[20] Meškytė EM, Keskas S, Ciribilli Y. MYC as a multifaceted regulator of tumor microenvironment leading to metastasis. Int J Mol Sci. 2020. 10.3390/ijms21207710. 33081056 10.3390/ijms21207710 PMC7589112

[21] Liu B, Zhang X. Metabolic reprogramming underlying brain metastasis of breast cancer. Front Mol Biosci. 2021;8:791927. 35071325 10.3389/fmolb.2021.791927 PMC8766845

[22] Lee HY, Cha J, Kim SK, Park JH, Song KH, Kim P, et al. c-MYC drives breast cancer metastasis to the brain, but promotes synthetic lethality with TRAIL. Mol Cancer Res. 2019;17(2):544–54. 30266755 10.1158/1541-7786.MCR-18-0630

[23] Van Swearingen AED, Lee MR, Rogers LW, Sibley AB, Shi P, Qin X, et al. Genomic and immune profiling of breast cancer brain metastases. Acta Neuropathol Commun. 2025;13(1):99. 40355907 10.1186/s40478-025-02001-3 PMC12070617

[24] Garralda E, Beaulieu ME, Moreno V, Casacuberta-Serra S, Martínez-Martín S, Foradada L, et al. MYC targeting by OMO-103 in solid tumors: a phase 1 trial. Nat Med. 2024;30(3):762–71. 38321218 10.1038/s41591-024-02805-1 PMC10957469

[25] Li K, Wang B, Hu H. Research progress of SWI/SNF complex in breast cancer. Epigenetics & Chromatin. 2024;17(1):4. 38365747 10.1186/s13072-024-00531-z PMC10873968

[26] Cheng X, Zhao JX, Dong F, Cao XC. ARID1A mutation in metastatic breast cancer: a potential therapeutic target. Front Oncol. 2021;11:759577. 34804958 10.3389/fonc.2021.759577 PMC8599951

[27] Wang T, Gao X, Zhou K, Jiang T, Gao S, Liu P, et al. Role of ARID1A in epithelial–mesenchymal transition in breast cancer and its effect on cell sensitivity to 5–FU. Int J Mol Med. 2020;46(5):1683–94. 33000179 10.3892/ijmm.2020.4727 PMC7521577

[28] Nagarajan S, Rao SV, Sutton J, Cheeseman D, Dunn S, Papachristou EK, et al. ARID1A influences HDAC1/BRD4 activity, intrinsic proliferative capacity and breast cancer treatment response. Nat Genet. 2020;52(2):187–97. 31913353 10.1038/s41588-019-0541-5 PMC7116647

[29] Phase A. 1/2a, Multi-Center, Open-Label Study to Evaluate the Safety, Tolerability, Pharmacokinetics, and Preliminary Evidence of Antitumor Activity of JAB-2485 in Adult Patients With Advanced Solid Tumors [Internet]. 2022. Available from: https://clinicaltrials.gov/study/NCT05490472

[30] Phase A. II Clinical Trial to Investigate ARID1A Mutation and CXCL13 Expression in the Pre-Treatment Tumor Samples as a Combinatorial Predictive Biomarker for Immune Checkpoint Therapy in Metastatic Solid Tumors [Internet]. 2021. Available from: https://clinicaltrials.gov/study/NCT04957615

[31] Ippen FM, Alvarez-Breckenridge CA, Kuter BM, Fink AL, Bihun IV, Lastrapes M, et al. The dual PI3K/mTOR pathway inhibitor GDC-0084 achieves antitumor activity in PIK3CA-Mutant breast cancer brain metastases. Clin Cancer Res. 2019;25(11):3374–83. 30796030 10.1158/1078-0432.CCR-18-3049 PMC6685218

[32] Peddi PF, Hurvitz SA. PI3K pathway inhibitors for the treatment of brain metastases with a focus on HER2 + breast cancer. J Neurooncol. 2014;117(1):7–13. 24469856 10.1007/s11060-014-1369-6 PMC3980725

[33] Laplante M, Sabatini DM. mTOR signaling in growth control and disease. Cell. 2012;149(2):274–93. 22500797 10.1016/j.cell.2012.03.017 PMC3331679

[34] Costa RLB, Han HS, Gradishar WJ. Targeting the PI3K/AKT/mTOR pathway in triple-negative breast cancer: a review. Breast Cancer Res Treat. 2018;169(3):397–406. 29417298 10.1007/s10549-018-4697-y

[35] Martinez-Outschoorn UE, Peiris-Pagés M, Pestell RG, Sotgia F, Lisanti MP. Cancer metabolism: a therapeutic perspective. Nat Rev Clin Oncol. 2017;14(2):113. 28094266 10.1038/nrclinonc.2017.1

[36] Saxton RA, Sabatini DM. mTOR signaling in growth, metabolism, and disease. Cell. 2017;169(2):361–71. 28388417 10.1016/j.cell.2017.03.035

[37] Brastianos PK, Carter SL, Santagata S, Cahill DP, Taylor-Weiner A, Jones RT, et al. Genomic characterization of brain metastases reveals branched evolution and potential therapeutic targets. Cancer Discov. 2015;5(11):1164–77. 26410082 10.1158/2159-8290.CD-15-0369 PMC4916970

[38] Open-label. Multicentre, Phase Ib Dose-escalation Study of MEN1611, a PI3K Inhibitor Combined With Trastuzumab With or Without Fulvestrant, in Subjects With PIK3CA Mutated HER2 Positive Locally Recurrent Unresectable (Advanced) or Metastatic (a/m) Breast Cancer Progressed to Anti-HER2 Based Therapy [Internet]. 2018. Available from: https://clinicaltrials.gov/study/NCT03767335

[39] Phase II Study for Patients With Advanced Triple Negative or Metaplastic HR-positive/HER2-negative, PIK3CA/PTEN-altered Breast Cancer Treated With Eribulin in Combination With MEN1611 [Internet]. 2023. Available from: https://clinicaltrials.gov/study/NCT05810870

[40] Jagust P, Powell AM, Ola M, Watson L, de Pablos-Aragoneses A, García-Gómez P, et al. RET overexpression leads to increased brain metastatic competency in luminal breast cancer. J Natl Cancer Inst. 2024;116(10):1632–44. 38852945 10.1093/jnci/djae091 PMC11461165

[41] Gattelli A, Nalvarte I, Boulay A, Roloff TC, Schreiber M, Carragher N, et al. Ret inhibition decreases growth and metastatic potential of estrogen receptor positive breast cancer cells. EMBO Mol Med. 2013;5(9):1335–50. 23868506 10.1002/emmm.201302625 PMC3799490

[42] Zhou C, Solomon B, Loong HH, Park K, Pérol M, Arriola E, et al. First-Line Selpercatinib or chemotherapy and pembrolizumab in RET Fusion-Positive NSCLC. N Engl J Med. 2023;389(20):1839–50. 37870973 10.1056/NEJMoa2309457 PMC10698285

[43] Subbiah V, Gainor JF, Oxnard GR, Tan DSW, Owen DH, Cho BC, et al. Intracranial efficacy of Selpercatinib in RET Fusion-Positive Non-Small cell lung cancers on the LIBRETTO-001 trial. Clin Cancer Res. 2021;27(15):4160–7. 34088726 10.1158/1078-0432.CCR-21-0800 PMC8447251

[44] Gao L, Zhang Y, Feng M, Shen M, Yang L, Wei B, et al. HER3: updates and current biology function, targeted therapy and pathologic detecting methods. Life Sci. 2024;357:123087. 39366553 10.1016/j.lfs.2024.123087

[45] Momeny M, Saunus JM, Marturana F, McCart Reed AE, Black D, Sala G, et al. Heregulin-HER3-HER2 signaling promotes matrix metalloproteinase-dependent blood-brain-barrier transendothelial migration of human breast cancer cell lines. Oncotarget. 2015;6(6):3932–46. 25668816 10.18632/oncotarget.2846 PMC4414164

[46] Zhu M, Yu M, Meng Y, Yang J, Wang X, Li L, et al. HER3 receptor and its role in the therapeutic management of metastatic breast cancer. J Transl Med. 2024;22(1):665. 39020378 10.1186/s12967-024-05445-8 PMC11253420

[47] Phase 1b/2, Multicenter, Open-label, Dose-Expansion Modular Study To Explore the Safety, Tolerability, and Anti-tumor Activity of HER3- DXd Monotherapy and Combinations in Patients With Inoperable Advanced Breast Cancer (ABC) After Progression on T-DXd [Internet]. 2024. Available from: https://clinicaltrials.gov/study/NCT06298084

[48] HERTHENA-PanTumor01 (U31402-277): A Phase 2, Multicenter, Multicohort, Open-Label, Proof of Concept Study of Patritumab Deruxtecan (HER3-DXd; U3-1402) in Subjects With Locally Advanced or Metastatic Solid Tumors [Internet]. 2023. Available from: https://clinicaltrials.gov/study/NCT06172478

[49] A Phase IIa Study of Dendritic Cell Vaccines Against Her2/Her3 and Pembrolizumab in Patients With Asymptomatic Brain Metastasis From Triple Negative Breast Cancer (TNBC) or HER2 + Breast Cancer (HER2 + BC) or Hormone Receptor Positive (HR+) Breast Cancer [Internet]. 2020. Available from: https://clinicaltrials.gov/study/NCT04348747

[50] NICE. Advanced breast cancer: diagnosis and treatment: NICE. 2009 [updated 19 February 2025. Available from: https://www.nice.org.uk/guidance/cg81/chapter/recommendations#diagnosis-and-assessment

[51] Derks SH, van der Veldt AA, Smits M. Brain metastases: the role of clinical imaging. Br J Radiol. 2022;95(1130):20210944. 34808072 10.1259/bjr.20210944 PMC8822566

[52] Roy-O’Reilly MA, Lanman T, Ruiz A, Rogawski D, Stocksdale B, Nagpal S. Diagnostic and therapeutic updates in leptomeningeal disease. Curr Oncol Rep. 2023;25(8):937–50. 37256537 10.1007/s11912-023-01432-2 PMC10326117

[53] Glass JP, Melamed M, Chernik NL, Posner JB. Malignant cells in cerebrospinal fluid (CSF): the meaning of a positive CSF cytology. Neurology. 1979;29(10):1369–75. 573381 10.1212/wnl.29.10.1369

[54] Cristofanilli M, Pierga JY, Reuben J, Rademaker A, Davis AA, Peeters DJ, et al. The clinical use of circulating tumor cells (CTCs) enumeration for staging of metastatic breast cancer (MBC): international expert consensus paper. Crit Rev Oncol Hematol. 2019;134:39–45. 30771872 10.1016/j.critrevonc.2018.12.004

[55] Darlix A, Cayrefourcq L, Pouderoux S, de Menjot Champfleur N, Bievelez A, Jacot W, et al. Detection of circulating tumor cells in cerebrospinal fluid of patients with suspected breast cancer leptomeningeal metastases: a prospective study. Clin Chem. 2022;68(10):1311–22. 35953885 10.1093/clinchem/hvac127

[56] Le Rhun E, Massin F, Tu Q, Bonneterre J, Bittencourt Mde C, Faure GC. Development of a new method for identification and quantification in cerebrospinal fluid of malignant cells from breast carcinoma leptomeningeal metastasis. BMC Clin Pathol. 2012;12:21. 23145812 10.1186/1472-6890-12-21 PMC3539901

[57] Riethdorf S, Fritsche H, Müller V, Rau T, Schindlbeck C, Rack B, et al. Detection of Circulating tumor cells in peripheral blood of patients with metastatic breast cancer: a validation study of the cellsearch system. Clin Cancer Res. 2007;13(3):920–8. 17289886 10.1158/1078-0432.CCR-06-1695

[58] Boire A, Brandsma D, Brastianos PK, Le Rhun E, Ahluwalia M, Junck L, et al. Liquid biopsy in central nervous system metastases: a RANO review and proposals for clinical applications. Neuro Oncol. 2019;21(5):571–84. 30668804 10.1093/neuonc/noz012 PMC6502489

[59] Jin Y, Zuo Y, Li G, Liu W, Pan Y, Fan T, et al. Advances in spatial transcriptomics and its applications in cancer research. Mol Cancer. 2024;23(1):129. 38902727 10.1186/s12943-024-02040-9 PMC11188176

[60] Tashjian RS, Vinters HV, Yong WH. Biobanking of cerebrospinal fluid. In: Biobanking: Methods and Protocols. 2018. p. 107–14. 10.1007/978-1-4939-8935-5_11 PMC6918832 30539439

[61] Tumani H, Petereit H, Gerritzen A, Gross C, Huss A, Isenmann S, et al. S1 guidelines “lumbar puncture and cerebrospinal fluid analysis”(abridged and translated version). Neurol Res Pract. 2020;2:1–28. 33324914 10.1186/s42466-020-0051-z PMC7650145

[62] McEwen AE, Leary SE, Lockwood CM. Beyond the blood: CSF-derived cfDNA for diagnosis and characterization of CNS tumors. Front Cell Dev Biol. 2020;8:45. 32133357 10.3389/fcell.2020.00045 PMC7039816

[63] Morganti S, Parsons HA, Lin NU, Grinshpun A. Liquid biopsy for brain metastases and leptomeningeal disease in patients with breast cancer. NPJ Breast Cancer. 2023;9(1):43. 37225714 10.1038/s41523-023-00550-1 PMC10209176

[64] Klotz R, Yu M. Insights into brain metastasis: recent advances in circulating tumor cell research. Cancer Rep. 2022;5(4):e1239. 10.1002/cnr2.1239 PMC9124503 33372393

[65] Li B, Zhang W, Guo C, Xu H, Li L, Fang M, et al. Benchmarking spatial and single-cell transcriptomics integration methods for transcript distribution prediction and cell type deconvolution. Nat Methods. 2022;19(6):662–70. 35577954 10.1038/s41592-022-01480-9

[66] Shapiro E, Biezuner T, Linnarsson S. Single-cell sequencing-based technologies will revolutionize whole-organism science. Nat Rev Genet. 2013;14(9):618–30. 23897237 10.1038/nrg3542

[67] Yoo J, editor Genetic, landscape of brain metastasis in triple-negative-breast cancer: Comparison of paired primary and metastatic tumor using spatial transcriptomic analysis. Neuro-Oncology. 2024: Oxford Univ Press Inc Journals Dept, 2001 Evans RD, CARY, NC 27513 USA.

[68] An J, Lu Y, Chen Y, Chen Y, Zhou Z, Chen J, et al. Spatial transcriptomics in breast cancer: providing insight into tumor heterogeneity and promoting individualized therapy. Front Immunol. 2024;15:1499301. 39749323 10.3389/fimmu.2024.1499301 PMC11693744

[69] Zhu S, Zhang M, Liu X, Luo Q, Zhou J, Song M, et al. Single-cell transcriptomics provide insight into metastasis-related subsets of breast cancer. Breast Cancer Res. 2023;25(1):126. 37858183 10.1186/s13058-023-01728-y PMC10588105

[70] Huang S, Ouyang L, Tang J, Qian K, Chen X, Xu Z, et al. Spatial transcriptomics: a new frontier in cancer research. Clin Cancer Bull. 2024;3(1):13.

[71] Dowling GP, Keelan S, Cosgrove NS, Daly GR, Giblin K, Toomey S, et al. Receptor discordance in metastatic breast cancer; a review of clinical and genetic subtype alterations from primary to metastatic disease. Breast Cancer Res Treat. 2024;207(3):471–6. 39090418 10.1007/s10549-024-07431-6 PMC11420314

[72] Schrijver WA, Suijkerbuijk KP, Van Gils CH, Van Der Wall E, Moelans CB, Van Diest PJ. Receptor conversion in distant breast cancer metastases: a systematic review and meta-analysis. J Natl Cancer Inst. 2018;110(6):568–80. 29315431 10.1093/jnci/djx273

[73] Aurilio G, Disalvatore D, Pruneri G, Bagnardi V, Viale G, Curigliano G, et al. A meta-analysis of oestrogen receptor, progesterone receptor and human epidermal growth factor receptor 2 discordance between primary breast cancer and metastases. Eur J Cancer. 2014;50(2):277–89. 24269135 10.1016/j.ejca.2013.10.004

[74] Grinda T, Joyon N, Lusque A, Lefèvre S, Arnould L, Penault-Llorca F, et al. Phenotypic discordance between primary and metastatic breast cancer in the large-scale real-life multicenter French ESME cohort. NPJ Breast Cancer. 2021;7(1):41. 33863896 10.1038/s41523-021-00252-6 PMC8052407

[75] Hulsbergen AFC, Claes A, Kavouridis VK, Ansaripour A, Nogarede C, Hughes ME, et al. Subtype switching in breast cancer brain metastases: a multicenter analysis. Neuro Oncol. 2020;22(8):1173–81. 31970416 10.1093/neuonc/noaa013 PMC7471502

[76] Venur VA, Cohen JV, Brastianos PK. Targeting molecular pathways in intracranial metastatic disease. Front Oncol. 2019;9:99. 30886831 10.3389/fonc.2019.00099 PMC6409309

[77] Shipitsin M, Campbell LL, Argani P, Weremowicz S, Bloushtain-Qimron N, Yao J, et al. Molecular definition of breast tumor heterogeneity. Cancer Cell. 2007;11(3):259–73. 17349583 10.1016/j.ccr.2007.01.013

[78] Criscitiello C, André F, Thompson AM, De Laurentiis M, Esposito A, Gelao L, et al. Biopsy confirmation of metastatic sites in breast cancer patients: clinical impact and future perspectives. Breast Cancer Res. 2014;16:1–8. 10.1186/bcr3630 PMC4052940 25032257

[79] Navin N, Kendall J, Troge J, Andrews P, Rodgers L, McIndoo J, et al. Tumour evolution inferred by single-cell sequencing. Nature. 2011;472(7341):90–4. 21399628 10.1038/nature09807 PMC4504184

[80] Broom RJ, Tang PA, Simmons C, Bordeleau L, Mulligan AM, O’MALLEY FP, et al. Changes in Estrogen receptor, progesterone receptor and Her-2/neu status with time: discordance rates between primary and metastatic breast cancer. Anticancer Res. 2009;29(5):1557–62. 19443366

[81] Karlsson E, Appelgren J, Solterbeck A, Bergenheim M, Alvariza V, Bergh J. Breast cancer during follow-up and progression–a population based cohort on new cancers and changed biology. Eur J Cancer. 2014;50(17):2916–24. 25241230 10.1016/j.ejca.2014.08.014

[82] Mittendorf EA, Wu Y, Scaltriti M, Meric-Bernstam F, Hunt KK, Dawood S, et al. Loss of HER2 amplification following trastuzumab-based neoadjuvant systemic therapy and survival outcomes. Clin Cancer Res. 2009;15(23):7381–8. 19920100 10.1158/1078-0432.CCR-09-1735 PMC2788123

[83] Pertschuk LP, Axiotis CA, Feldman JG, Kim YD, Karavattayhayyil SJ, Braithwaite L. Marked intratumoral heterogeneity of the proto-oncogene Her‐2/neu determined by three different detection systems. Breast J. 1999;5(6):369–74. 11348316 10.1046/j.1524-4741.1999.97088.x

[84] Thomson A, McGrane J, Mathew J, Palmer J, Hilton D, Purvis G, et al. Changing molecular profile of brain metastases compared with matched breast primary cancers and impact on clinical outcomes. Br J Cancer. 2016;114(7):793–800. 26908328 10.1038/bjc.2016.34 PMC4984859

[85] Dowling GP, Daly GR, Hegarty A, Flanagan M, Ola M, Fallon R et al. Neoadjuvant HER2 inhibition induces ESR1 DNA methylation alterations resulting in clinically relevant ER expression changes in breast cancers. Cancer Commun. 10.1002/cac2.12640 PMC11833670 39676516

[86] Almstedt K, Krauthauser L, Kappenberg F, Wagner DC, Heimes AS, Battista MJ, et al. Discordance of HER2-low between primary tumors and matched distant metastases in breast cancer. Cancers (Basel). 2023. 10.3390/cancers15051413. 36900203 10.3390/cancers15051413 PMC10000561

[87] Chambers AF, Naumov GN, Vantyghem SA, Tuck AB. Molecular biology of breast cancer metastasis clinical implications of experimental studies on metastatic inefficiency. Breast Cancer Res. 2000;2:1–8. 10.1186/bcr86 PMC138662 11250733

[88] Shah SP, Morin RD, Khattra J, Prentice L, Pugh T, Burleigh A, et al. Mutational evolution in a lobular breast tumour profiled at single nucleotide resolution. Nature. 2009;461(7265):809–13. 19812674 10.1038/nature08489

[89] Amir E, Miller N, Geddie W, Freedman O, Kassam F, Simmons C, et al. Prospective study evaluating the impact of tissue confirmation of metastatic disease in patients with breast cancer. J Clin Oncol. 2012;30(6):587–92. 22124102 10.1200/JCO.2010.33.5232 PMC5015424

[90] Thompson AM, Jordan LB, Quinlan P, Anderson E, Skene A, Dewar JA, et al. Prospective comparison of switches in biomarker status between primary and recurrent breast cancer: the breast recurrence in tissues study (BRITS). Breast Cancer Res. 2010;12:1–9. 10.1186/bcr2771 PMC3046433 21059212

[91] Vogelbaum MA, Brown PD, Messersmith H, Brastianos PK, Burri S, Cahill D, et al. Treatment for brain metastases: ASCO-SNO-ASTRO guideline. Oxford University Press US; 2022. 10.1200/JCO.21.02314 34932393

[92] Le Rhun E, Guckenberger M, Smits M, Dummer R, Bachelot T, Sahm F, et al. EANO–ESMO clinical practice guidelines for diagnosis, treatment and follow-up of patients with brain metastasis from solid tumours. Ann Oncol. 2021;32(11):1332–47. 34364998 10.1016/j.annonc.2021.07.016

[93] Van Grinsven EE, Nagtegaal SH, Verhoeff JJ, Van Zandvoort MJ. The impact of stereotactic or whole brain radiotherapy on neurocognitive functioning in adult patients with brain metastases: a systematic review and meta-analysis. Oncol Res Treat. 2021;44(11):622–36. 34482312 10.1159/000518848 PMC8686730

[94] Shang W, Yao H, Sun Y, Mu A, Zhu L, Li X. Preventive effect of hippocampal sparing on cognitive dysfunction of patients undergoing whole-brain radiotherapy and imaging assessment of hippocampal volume changes. Biomed Res Int. 2022;2022(1):4267673. 35425838 10.1155/2022/4267673 PMC9005304

[95] Hippocampal Sparing Whole Brain Radiation Versus Stereotactic Radiation (SRS) in Patients With 5–20 Brain Metastases: A Phase III, Randomized Clinical Trial [Internet]. 2017. Available from: https://clinicaltrials.gov/study/NCT03075072

[96] Prabhu RS, Press RH, Patel KR, Boselli DM, Symanowski JT, Lankford SP, et al. Single-fraction stereotactic radiosurgery (SRS) alone versus surgical resection and SRS for large brain metastases: a multi-institutional analysis. Int J Radiation Oncology* Biology* Phys. 2017;99(2):459–67. 10.1016/j.ijrobp.2017.04.006 28871997

[97] Borm KJ, Behzadi ST, Hörner-Rieber J, Krug D, Baumann R, Corradini S, et al. DEGRO guideline for personalized radiotherapy of brain metastases and leptomeningeal carcinomatosis in patients with breast cancer. Strahlenther Onkol. 2024;200(4):259–75. 38488902 10.1007/s00066-024-02202-0 PMC10965583

[98] Melisko ME, Assefa M, Hwang J, DeLuca A, Park JW, Rugo HS. Phase II study of irinotecan and temozolomide in breast cancer patients with progressing central nervous system disease. Breast Cancer Res Treat. 2019;177:401–8. 31172405 10.1007/s10549-019-05309-6

[99] Lin NU, Murthy RK, Abramson V, Anders C, Bachelot T, Bedard PL, et al. Tucatinib vs placebo, both in combination with trastuzumab and capecitabine, for previously treated ERBB2 (HER2)-positive metastatic breast cancer in patients with brain metastases: updated exploratory analysis of the HER2CLIMB randomized clinical trial. JAMA Oncol. 2023;9(2):197–205. 36454580 10.1001/jamaoncol.2022.5610 PMC9716438

[100] Nabors LB, Portnow J, Ahluwalia M, Baehring J, Brem H, Brem S, et al. Central nervous system cancers, version 3.2020, NCCN clinical practice guidelines in oncology. J Natl Compr Canc Netw. 2020;18(11):1537–70. 33152694 10.6004/jnccn.2020.0052

[101] Bartsch R, Berghoff AS, Furtner J, Marhold M, Bergen ES, Roider-Schur S, et al. Final outcome analysis from the phase II TUXEDO-1 trial of trastuzumab-deruxtecan in HER2-positive breast cancer patients with active brain metastases. Neurooncology. 2024;26(12):2305–15. 10.1093/neuonc/noae123 PMC11630562 38963808

[102] Harbeck N, Ciruelos E, Jerusalem G, Müller V, Niikura N, Viale G, et al. Trastuzumab Deruxtecan in HER2-positive advanced breast cancer with or without brain metastases: a phase 3b/4 trial. Nat Med. 2024;30(12):3717–27. 39271844 10.1038/s41591-024-03261-7 PMC11645283

[103] Tolaney SM, Jiang Z, Zhang Q, Barroso-Sousa R, Park YH, Rimawi MF et al. Trastuzumab deruxtecan plus pertuzumab for HER2-positive metastatic breast cancer. New Engl J Med. 0(0). 10.1056/NEJMoa2508668 41160818

[104] Wang W, He H, Marín-Ramos NI, Zeng S, Swenson SD, Cho HY, et al. Enhanced brain delivery and therapeutic activity of trastuzumab after blood-brain barrier opening by NEO100 in mouse models of brain-metastatic breast cancer. Neuro Oncol. 2021;23(10):1656–67. 33659980 10.1093/neuonc/noab041 PMC8485439

[105] Shah M, Wedam S, Cheng J, Fiero MH, Xia H, Li F, et al. FDA approval summary: tucatinib for the treatment of patients with advanced or metastatic HER2-positive breast cancer. Clin Cancer Res. 2021;27(5):1220–6. 33055172 10.1158/1078-0432.CCR-20-2701

[106] Murthy RK, Loi S, Okines A, Paplomata E, Hamilton E, Hurvitz SA, et al. Tucatinib, Trastuzumab, and Capecitabine for HER2-positive metastatic breast cancer. N Engl J Med. 2020;382(7):597–609. 31825569 10.1056/NEJMoa1914609

[107] Gouveia MC, Hidalgo Filho CM, Moreno RA, Alves H, Ayres AS, Testa L, et al. Activity of capecitabine for central nervous system metastases from breast cancer. Ecancermedicalscience. 2023;17:1638. 38414937 10.3332/ecancer.2023.1638 PMC10898896

[108] Saura C, Oliveira M, Feng YH, Dai MS, Chen SW, Hurvitz SA, et al. Neratinib plus capecitabine versus lapatinib plus capecitabine in HER2-Positive metastatic breast cancer previously treated with ≥ 2 HER2-Directed regimens: phase III NALA trial. J Clin Oncol. 2020;38(27):3138–49. 32678716 10.1200/JCO.20.00147 PMC7499616

[109] Hurvitz SA, Saura C, Oliveira M, Trudeau ME, Moy B, Delaloge S, et al. Efficacy of neratinib plus capecitabine in the subgroup of patients with central nervous system involvement from the NALA trial. Oncologist. 2021;26(8):e1327–38. 34028126 10.1002/onco.13830 PMC8342591

[110] Wuerstlein R, Ellis P, Montemurro F, Antón Torres A, Delaloge S, Zhang Q, et al. Final results of the global and Asia cohorts of KAMILLA, a phase IIIB safety trial of trastuzumab emtansine in patients with HER2-positive advanced breast cancer. ESMO Open. 2022;7(5):100561. 36084395 10.1016/j.esmoop.2022.100561 PMC9588895

[111] A Phase II Study of Tucatinib and Ado-trastuzumab Emtansine (T-DM1) in Patients With HER2-positive Metastatic Solid Tumors and Metastases to Brain (TUCATEMEB) [Internet]. 2022. Available from: https://clinicaltrials.gov/study/NCT05673928

[112] Montemurro F, Delaloge S, Barrios CH, Wuerstlein R, Anton A, Brain E, et al. Trastuzumab emtansine (T-DM1) in patients with HER2-positive metastatic breast cancer and brain metastases: exploratory final analysis of cohort 1 from KAMILLA, a single-arm phase IIIb clinical trial(☆). Ann Oncol. 2020;31(10):1350–8. 32634611 10.1016/j.annonc.2020.06.020

[113] Curtaz CJ, Kiesel L, Meybohm P, Wöckel A, Burek M. Anti-hormonal therapy in breast cancer and its effect on the blood-brain barrier. Cancers (Basel). 2022. 10.3390/cancers14205132. 36291916 10.3390/cancers14205132 PMC9599962

[114] Conlan MG, de Vries EFJ, Glaudemans A, Wang Y, Troy S. Pharmacokinetic and Pharmacodynamic Studies of Elacestrant, A Novel Oral Selective Estrogen Receptor Degrader, in Healthy Post-Menopausal Women. Eur J Drug Metab Pharmacokinet. 2020;45(5):675–89. 32661909 10.1007/s13318-020-00635-3 PMC7511284

[115] Sarfraz A, Sarfraz M, Javad F, Khalid M, Shah B, Gul A, et al. Elacestrant in hormone receptor-positive metastatic breast cancer: a post-hoc analysis. Explor Target Antitumor Ther. 2025;6:1002293. 39991467 10.37349/etat.2025.1002293 PMC11847623

[116] Phase A. IB Study to evaluate the effect of RAD1901 on the availability of estrogen receptor binding sites in metastatic breast cancer lesions using 16α-18F-Fluoro-17β-Estradiol positron emission tomography imaging [Internet]. 2016. Available from: https://clinicaltrials.gov/study/NCT02650817

[117] Phase A, Multicenter I, Open-Label. Multi-Part, Dose-escalation Study of RAD1901 in postmenopausal women with advanced estrogen receptor positive and HER2-negative breast cancer [Internet]. 2014. Available from: https://clinicaltrials.gov/study/NCT02338349

[118] Jhaveri KL, Neven P, Casalnuovo ML, Kim S-B, Tokunaga E, Aftimos P, et al. Imlunestrant with or without abemaciclib in advanced breast cancer. N Engl J Med. 2025;392(12):1189–202.39660834 10.1056/NEJMoa2410858

[119] Chabot P, Hsia TC, Ryu JS, Gorbunova V, Belda-Iniesta C, Ball D, et al. Veliparib in combination with whole-brain radiation therapy for patients with brain metastases from non-small cell lung cancer: results of a randomized, global, placebo-controlled study. J Neurooncol. 2017;131(1):105–15. 27655223 10.1007/s11060-016-2275-x PMC5258788

[120] Loibl S, O’Shaughnessy J, Untch M, Sikov WM, Rugo HS, McKee MD, et al. Addition of the PARP inhibitor veliparib plus carboplatin or carboplatin alone to standard neoadjuvant chemotherapy in triple-negative breast cancer (BrighTNess): a randomised, phase 3 trial. Lancet Oncol. 2018;19(4):497–509. 29501363 10.1016/S1470-2045(18)30111-6

[121] Tung NM, Robson ME, Ventz S, Santa-Maria CA, Nanda R, Marcom PK, et al. TBCRC 048: phase II study of olaparib for metastatic breast cancer and mutations in homologous recombination-related genes. J Clin Oncol. 2020;38(36):4274–82. 33119476 10.1200/JCO.20.02151

[122] Carruthers R, Chalmers AJ. The potential of PARP inhibitors in neuro-oncology. CNS Oncol. 2012;1(1):85–97. 25054302 10.2217/cns.12.13 PMC6176825

[123] Giordano A, Kumthekar PU, Jin Q, Binboga Kurt B, Ren S, Li T, et al. A phase II study of atezolizumab, pertuzumab, and high-dose trastuzumab for central nervous system metastases in patients with HER2-positive breast cancer. Clin Cancer Res. 2024;30(21):4856–65. 39226397 10.1158/1078-0432.CCR-24-1161 PMC11528201

[124] Phase A. II Study of atezolizumab in combination with pertuzumab plus high-dose trastuzumab for the treatment of central nervous system metastases in patients with Her2-positive breast cancer [Internet]. 2018. Available from: https://clinicaltrials.gov/study/NCT03417544

[125] Adebrelimab Plus Apatinib and Etoposide for the Treatment of HER2-Negative Breast Cancer Brain Metastasis: A Phase II Study [Internet]. 2024. Available from: https://clinicaltrials.gov/study/NCT06418594

[126] Zhou S, Xie J, Huang Z, Deng L, Wu L, Yu J, et al. Anti-PD-(L)1 immunotherapy for brain metastases in non-small cell lung cancer: mechanisms, advances, and challenges. Cancer Lett. 2021;502:166–79. 33450361 10.1016/j.canlet.2020.12.043

[127] Yuzhalin AE, Lowery FJ, Saito Y, Yuan X, Yao J, Duan Y, et al. Astrocyte-induced Cdk5 expedites breast cancer brain metastasis by suppressing MHC-I expression to evade immune recognition. Nat Cell Biol. 2024;26(10):1773–89. 39304713 10.1038/s41556-024-01509-5 PMC11676029

[128] Li H, Wang J, Yi Z, Li C, Wang H, Zhang J, et al. CDK12 Inhibition enhances sensitivity of HER2 + breast cancers to HER2-tyrosine kinase inhibitor via suppressing PI3K/AKT. Eur J Cancer. 2021;145:92–108. 33429148 10.1016/j.ejca.2020.11.045

[129] Modular A, Multi-Part M-A. Phase 1/2 Study to evaluate the safety and tolerability of CT7439 alone and in combination with anticancer treatments in participants with solid malignancies [Internet]. 2024. Available from: https://clinicaltrials.gov/study/NCT06600789

[130] Bhogal T, Giannoudis A, Sokol E, Ali S, Palmieri C. Analysis of breast cancer brain metastases reveals an enrichment of Cyclin-Dependent kinase 12 structural rearrangements in human epidermal growth factor receptor 2–Positive disease. JCO Precis Oncol. 2024;8:e2300639. 38838276 10.1200/PO.23.00639

[131] Morikawa A, Li J, Ulintz P, Cheng X, Apfel A, Robinson D, et al. Optimizing precision medicine for breast cancer brain metastases with functional drug response assessment. Cancer Res Commun. 2023;3(6):1093–103. 37377606 10.1158/2767-9764.CRC-22-0492 PMC10284082

[132] Bartsch R, Berghoff AS, Furtner J, Marhold M, Bergen ES, Roider-Schur S, et al. Trastuzumab Deruxtecan in HER2-positive breast cancer with brain metastases: a single-arm, phase 2 trial. Nat Med. 2022;28(9):1840–7. 35941372 10.1038/s41591-022-01935-8 PMC9499862

[133] Li C, Liu M, Zhang Y, Wang Y, Li J, Sun S, et al. Novel models by machine learning to predict prognosis of breast cancer brain metastases. J Transl Med. 2023;21(1):404. 37344847 10.1186/s12967-023-04277-2 PMC10286496

[134] Tan Y, Zhang W-h, Huang Z, Tan Q-x, Zhang Y-m, Wei C-y, et al. AI models predicting breast cancer distant metastasis using LightGBM with clinical blood markers and ultrasound maximum diameter. Sci Rep. 2024;14(1):15561. 38969798 10.1038/s41598-024-66658-x PMC11226620

[135] Shiner A, Kiss A, Saednia K, Jerzak KJ, Gandhi S, Lu FI, et al. Predicting patterns of distant metastasis in breast cancer patients following local regional therapy using machine learning. Genes. 2023. 10.3390/genes14091768. 37761908 10.3390/genes14091768 PMC10531341

[136] Grøvik E, Yi D, Iv M, Tong E, Rubin D, Zaharchuk G. Deep learning enables automatic detection and segmentation of brain metastases on multisequence MRI. J Magn Reson Imaging. 2020;51(1):175–82. 31050074 10.1002/jmri.26766 PMC7199496

[137] Ziyaee H, Cardenas CE, Yeboa DN, Li J, Ferguson SD, Johnson J, et al. Automated brain metastases segmentation with a deep dive into false-positive detection. Adv Radiat Oncol. 2023;8(1):101085. 36299565 10.1016/j.adro.2022.101085 PMC9589017

[138] Forghani R. Precision digital oncology: emerging role of radiomics-based biomarkers and artificial intelligence for advanced imaging and characterization of brain tumors. Radiology: Imaging Cancer. 2020;2(4):e190047. 33778721 10.1148/rycan.2020190047 PMC7983689

[139] Nowakowski A, Lahijanian Z, Panet-Raymond V, Siegel PM, Petrecca K, Maleki F, et al. Radiomics as an emerging tool in the management of brain metastases. Neuro-Oncology Adv. 2022;4(1):vdac141. 10.1093/noajnl/vdac141 PMC9583687 36284932

[140] Kocak B, Bulut E, Bayrak ON, Okumus AA, Altun O, Borekci Arvas Z, et al. NEgatiVE results in radiomics research (NEVER): A meta-research study of publication bias in leading radiology journals. Eur J Radiol. 2023;163:110830. 37119709 10.1016/j.ejrad.2023.110830

[141] Wang JY, Qu V, Hui C, Sandhu N, Mendoza MG, Panjwani N, et al. Stratified assessment of an FDA-cleared deep learning algorithm for automated detection and contouring of metastatic brain tumors in stereotactic radiosurgery. Radiat Oncol. 2023;18(1):61. 37016416 10.1186/s13014-023-02246-z PMC10074777

[142] Li Y, Liu Y, Liang Y, Wei R, Zhang W, Yao W, et al. Radiomics can differentiate high-grade glioma from brain metastasis: a systematic review and meta-analysis. Eur Radiol. 2022;32(11):8039–51. 35587827 10.1007/s00330-022-08828-x

[143] Bejarano L, Kauzlaric A, Lamprou E, Lourenco J, Fournier N, Ballabio M, et al. Interrogation of endothelial and mural cells in brain metastasis reveals key immune-regulatory mechanisms. Cancer Cell. 2024;42(3):378–95. e10. 38242126 10.1016/j.ccell.2023.12.018

[144] Wang Y, Ye F, Liang Y, Yang Q. Breast cancer brain metastasis: insight into molecular mechanisms and therapeutic strategies. Br J Cancer. 2021;125(8):1056–67. 34226684 10.1038/s41416-021-01424-8 PMC8505648

[145] Neman J, Termini J, Wilczynski S, Vaidehi N, Choy C, Kowolik CM, et al. Human breast cancer metastases to the brain display GABAergic properties in the neural niche. Proc Natl Acad Sci U S A. 2014;111(3):984–9. 24395782 10.1073/pnas.1322098111 PMC3903266

[146] Zeng Q, Michael IP, Zhang P, Saghafinia S, Knott G, Jiao W, et al. Synaptic proximity enables NMDAR signalling to promote brain metastasis. Nature. 2019;573(7775):526–31. 31534217 10.1038/s41586-019-1576-6 PMC6837873

[147] Chen Q, Boire A, Jin X, Valiente M, Er EE, Lopez-Soto A, et al. Carcinoma–astrocyte gap junctions promote brain metastasis by cGAMP transfer. Nature. 2016;533(7604):493–8. 27225120 10.1038/nature18268 PMC5021195

[148] Evans KT, Blake K, Longworth A, Coburn MA, Insua-Rodríguez J, McMullen TP, et al. Microglia promote anti-tumour immunity and suppress breast cancer brain metastasis. Nat Cell Biol. 2023;25(12):1848–59. 37957324 10.1038/s41556-023-01273-y PMC11414741

[149] Ruan X, Yan W, Cao M, Daza RAM, Fong MY, Yang K, et al. Breast cancer cell-secreted miR-199b-5p hijacks neurometabolic coupling to promote brain metastasis. Nat Commun. 2024;15(1):4549. 38811525 10.1038/s41467-024-48740-0 PMC11137082

